# Molecular recognition and effects of a benzothiazole derivative targeting the *MYC* G-quadruplex

**DOI:** 10.1093/nar/gkaf888

**Published:** 2025-09-12

**Authors:** Xiao Ni, Xiao-Dong Hu, Wei Long, Wenxian Lan, Chunxi Wang, Wing-Leung Wong, Chunyang Cao

**Affiliations:** State Key Laboratory of Chemical Biology, Shanghai Institute of Organic Chemistry, University of Chinese Academy of Sciences, Chinese Academy of Sciences, Shanghai 200032, China; State Key Laboratory of Chemical Biology, Shanghai Institute of Organic Chemistry, University of Chinese Academy of Sciences, Chinese Academy of Sciences, Shanghai 200032, China; State Key Laboratory of Chemical Biology and Drug Discovery, Department of Applied Biology and Chemical Technology, The Hong Kong Polytechnic University, Kowloon, Hong Kong SAR 999077, China; The Core Facility Center of CAS Center for Excellence in Molecular Plant Sciences, Institute of Plant Physiology and Ecology, Chinese Academy of Sciences, Shanghai 200032, China; State Key Laboratory of Chemical Biology, Shanghai Institute of Organic Chemistry, University of Chinese Academy of Sciences, Chinese Academy of Sciences, Shanghai 200032, China; State Key Laboratory of Chemical Biology and Drug Discovery, Department of Applied Biology and Chemical Technology, The Hong Kong Polytechnic University, Kowloon, Hong Kong SAR 999077, China; State Key Laboratory of Chemical Biology, Shanghai Institute of Organic Chemistry, University of Chinese Academy of Sciences, Chinese Academy of Sciences, Shanghai 200032, China

## Abstract

Small-molecule intervention and stabilization of G-quadruplexes (G4s) have been investigated for the potential as therapeutic approaches. MYC plays diverse roles in cellular functions, making it a highly desirable yet challenging target. One promising strategy includes DNA G4 structures, which mediate transcriptional control over MYC in the presence of small-molecule ligands. Unraveling the effects of these ligands on G4 stability and functionality is seldom achieved yet essential for designing potent ligands against these intractable targets. This study introduces BTO-28, a benzothiazole-based ligand that binds with high affinity to the *MYC* G4. *In vitro* experiments, NMR analysis, and intracellular assays collectively indicate that BTO-28 potentially downregulates MYC transcription through a G4-mediated mechanism. Structural determination of the 2:1 benzothiazole–*MYC* G4 complex provides insights into unexpected molecular interactions, highlighting for the first time a unique hydrogen-bonding pattern involving the nucleobase surrogate and flanking residues. The protonated pyrrolidine side chains of BTO-28 reorient to form hydrogen bonding with the external G-tetrad, establishing a previously uncharacterized ligand–G4 interface. This work advances the rational design of G4-binding ligands and clarifies the molecular basis underlying *MYC* recognition.

## Graphical Abstract

**Graphical Abstract:**
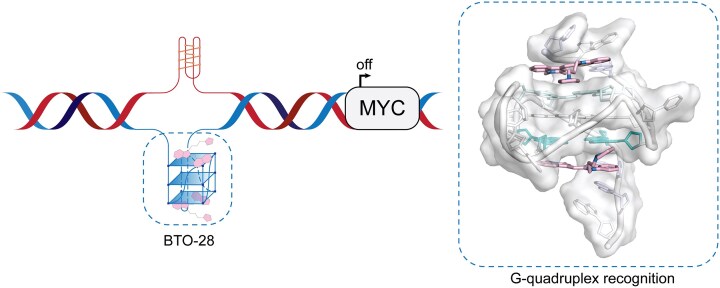

## Introduction

G-quadruplexes (G4s) are noncanonical four-stranded DNA structures that play regulatory roles in key genomic regions [1]. These structures originate from guanine-rich sequences under physiological conditions [2], and their folding is dynamic in live cells [3]. They are commonly located in human telomeres [4], the promoters of genes such as *MYC* [5, 6], *EGFR* [7], and *KIT* [8], as well as untranslated regions [9]. Using high-resolution techniques for genome-wide analysis, researchers have uncovered a more extensive and diverse set of G4 structures than previously recognized [10, 11]. G4s are emerging as promising molecular targets for therapeutic interventions, with notable potential in targeting *MYC* and additional oncogenes [12]. The oncogene *MYC* coordinates critical transcriptional programs involved in the initiation and maintenance of tumorigenesis [13]. Elevated expression of MYC is associated with more than half of human cancers through multiple mechanisms, including gene amplification, chromosomal translocation, and mutation of upstream signaling pathways [14]. Endogenous MYC is essential for maintenance of solid tumor and its inhibition has demonstrated potential therapeutic value [15]. MYC’s pleiotropic roles in diverse cellular processes making it a coveted therapeutic target, but several technical challenges, especially the intrinsically disordered binding region [16], have so far hindered the development of MYC inhibitory drugs. A significant amount of medicinal researches have focused on targeting MYC, employing a variety of strategies to address this challenging target [17, 18]. Noteworthy efforts include disruption of MYC-MAX heterodimerization, which inhibits E-box DNA binding and promotes the degradation of MYC [19, 20]. Recent entry of OMO-103 into clinical trials has demonstrated both safety and preliminary antitumor activity [21]. Further studies are ongoing to assess its therapeutic potential.

An alternative approach has been explored to perturb the transcriptional activity of MYC by targeting its promoter region. MYC dysregulation in most human cancers is typically not due to mutations in the *MYC* gene itself but rather a consequence of its activation by upstream oncogenic signaling pathways [22]. Negative supercoiling induced by *MYC* transcription has been shown to drive topological changes from duplex DNA to non-B-DNA structures [23]. Some transcription factors, such as SP1, activate transcription by binding to the duplex form of Nuclease Hypersensitive Element III_1_ (NHE III_1_) [24]. The formation of G-quadruplexes within this element sequesters SP1 and other proteins, consequently acting as a transcriptional repressor of MYC expression [25–27]. Genetic abrogation of the folding of endogenous G4 structures leads to a reduction in transcription factor recruitment in cells [28]. Endogenous G4 formation at gene promoters in live cells has been demonstrated using G4-selective antibodies and small-molecule probes [29]. However, the functional role of G4 in transcription remains unresolved. Molecules that selectively recognize distinct DNA folds in the human genome could be used to perturb gene expression or regulate signaling pathways [30–32], offering potential as therapeutic agents. Further credence has been given to the strategy of targeting G4 structures by the recent investigation of CX-5461 in phase I and II clinical trials for patients with *BRCA1*-/*BRCA2*-deficient tumors [33, 34]. Proposed mechanisms for selective inhibition of CX-5461 include stabilizing G4 structures and impeding replication fork progression [35]. The mutagenic effects of CX-5461 in normal cells raise safety concerns [33] and underscore the need for a deeper understanding of G4–ligand interactions in the drug development. To unravel the mechanistic role of G4 formation in gene regulation, it is essential to study G4-interacting ligands, especially those that may modulate gene expression.

**Figure 1.**
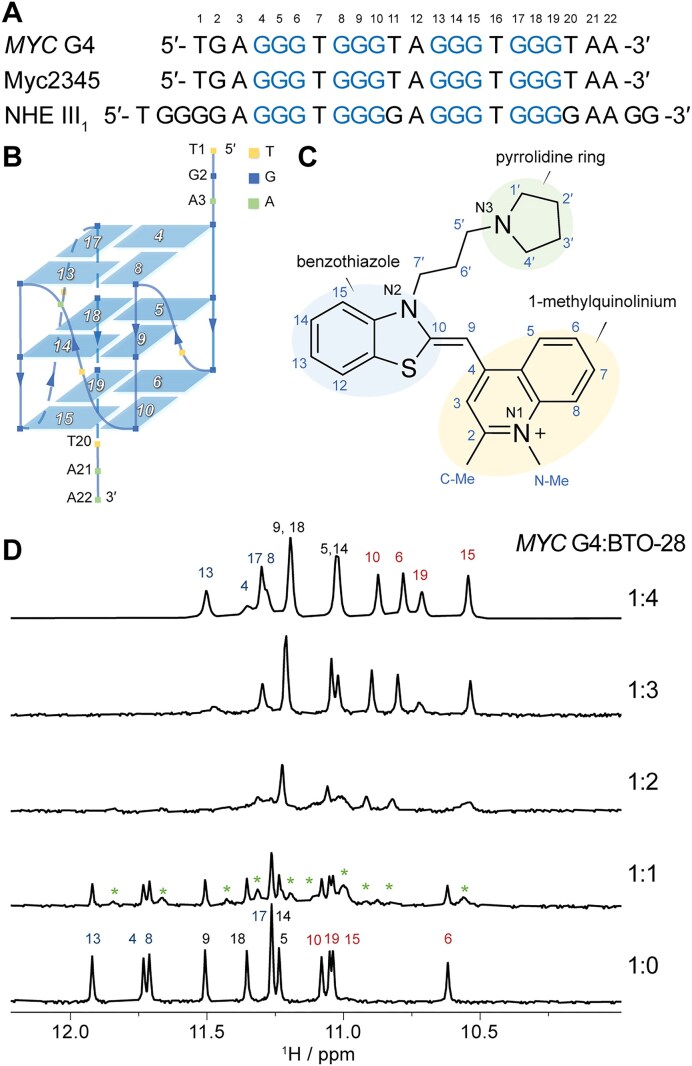
(**A**) The sequences of the NHE III_1_ element from the *MYC* promoter and its variants. (**B**) Schematic representation of the *MYC* G4 folding topology. (**C**) Chemical structure of the benzothiazole derivative BTO-28, with its atom numbering system. (**D**) Expanded imino region of 1D ^1^H NMR spectra during titration of BTO-28 into *MYC* G4 (100 μM) at pH 7.4 with 10 mM K^+^. The molar ratios of *MYC* G4 to BTO-28 are 1:0, 1:1, 1:2, 1:3, and 1:4. The imino protons are color-coded in each spectrum: blue for the 5′ G-tetrad, black for the middle G-tetrad, and red for the 3′ G-tetrad. New peaks arising from BTO-28–*MYC* G4 complexes are marked with green asterisks.

The *MYC* G-quadruplex, formed in the NHE III_1_ region of the *MYC* oncogene promoter (Fig. 1A), is essential for regulating multiple cellular processes [36]. Pioneering studies, including the work by Yang and coworkers, identified the major G4 structure of this regulatory element using nuclear magnetic resonance (NMR) spectroscopy [37], with subsequent validation by X-ray crystallography [38]. In a recent structural investigation, a platinum(II) compound was found to stabilize the *MYC* G-quadruplex in successive stages and inhibit *MYC* transcription [39]; the work highlights NMR as an effective method for probing ligand–G4 interactions. Our study focuses on the NHE III_1_ element from the *MYC* promoter and its variants. The wild-type NHE III_1_ is a 27-nucleotide sequence rich in guanine. The predominant *MYC* G4 structure adopts a parallel topology and comprises four consecutive 3′-runs of guanines, with G11 and G20 not contributing to the formation of the tetrads [37]. Myc2345 retains the wild-type guanine at position 20, while *MYC* G4 (Myc2345_T23) features a G-to-T substitution that limits the loop–isomer formation (Fig. 1B) [40]. The 2.6-Å structure of nucleolin bound to an NHE III_1_-derived *MYC* G4 highlights loop and flanking interactions essential for G4–specific recognition both biochemically and in cells [27].

Thiazole orange is often employed as a nucleobase surrogate [41, 42], demonstrating elevated fluorescence upon incorporation into DNA. This enhancement is likely due to constrained orientations imposed by nearby base pairs [43], though the exact binding mechanism remains uncertain. The fluorescence of the thiazole orange derivatives remains unaffected by the photoinduced electron transfer quenching typically triggered by guanine [44], thereby enhancing their feasibility of targeting guanine-rich DNA sequences. Recent studies have revealed that a thiazole orange scaffold with multisite binding system can visualize endogenous G-quadruplex structures [45, 46], yet the rationale for its structural specificity remains unknown. Visualizing G4–ligand complexes at the molecular level would provide insights for the rational design of potent G4-targeting agents.

Herein, we detail the unique intermolecular binding mode of BTO-28, a high-affinity ligand of the *MYC* G4 (Fig. 1C). An earlier preliminary investigation reported that this ligand does not exhibit selective binding to telomeric G-quadruplexes [46]. Using NMR spectroscopy, the solution structures of the ligand in complex with *MYC* G4 are unambiguously determined. BTO-28 functions as a base surrogate in this unimolecular G-quadruplex, a mechanism not previously reported. Instead of simply stacking atop the external G-tetrads, the functional groups of the ligand match the hydrogen-bond arrangement of the neighboring residues while also interact with loop residues. Notably, the observed decrease in *MYC* transcription appears to be regulated by the NHE III_1_ region through a G-quadruplex-related mechanism. Chemical and structural investigations provide molecular basis for the recognition and functional regulation of *MYC* G-quadruplex.

## Materials and methods

### Sample preparation

Unlabeled DNA sequences and 5′ or 3′-*Cy5* labeled oligonucleotides were obtained from Sangon Biotech. DNA samples were annealed by heating to 98°C for 10 min, followed by gradual cooling to room temperature. DNA concentration was quantified using a NanoDrop spectrophotometer (Thermo Fisher Scientific). BTO-28 was synthesized as previously described (Supplementary Figs S17 and S18) [46]. It was dissolved in dimethyl sulfoxide (DMSO) to prepare a 100 mM stock solution, then diluted to the specified working concentrations for the experiments. Water samples were prepared in a solution comprising 10% D_2_O and 90% H_2_O. Samples in D_2_O were prepared by lyophilization and followed by dissolution in 99.9% D_2_O (CIL).

### Mass spectroscopy

DNA samples (100 μM) were prepared in in 20 mM potassium phosphate buffer (pH 7.4) containing 80 mM KCl. The solution was heated to 98°C for 10 min, then allowed to cool slowly to room temperature. BTO-28 was added at the specified DNA:ligand ratios and incubated for 30 min. Analysis was conducted using high-performance liquid chromatography coupled to electrospray ionization mass spectrometer (HPLC–ESI–MS). Related data were processed using Xcalibur.

### Circular dichroism spectroscopy

Circular dichroism (CD) experiments were performed using a JASCO-815 spectrometer (JASCO, Inc.). Samples were prepared in 20 mM Tris–HCl buffer containing 80 mM KCl at pH 7.4, to a final DNA concentration of 100 μM. The solution was heated to 98°C for 10 min, then allowed to cool slowly to room temperature. BTO-28 was added at the specified DNA:ligand ratios and incubated for 30 min. A 10-mm quartz cuvette was used, and the spectra were obtained by averaging three scans across the wavelength range of 200–400 nm. Baseline correction was performed by subtracting the buffer spectrum.

### Fluorescence polarization assays

*Cy5*-labeled G-quadruplex constructs are prepared in 20 mM potassium phosphate buffer (pH 7.4) containing 80 mM KCl and annealed as described above. Ligand solutions are prepared by diluting compounds in the same buffer to final concentrations of 0–2 μM. To each well, G4 was added to a final concentration of 20 nM, and the plates were incubated at room temperature for 30 min. Fluorescence polarization was subsequently measured using an EnVision microplate reader (PerkinElmer), and binding affinities were derived from single-site binding model fits in GraphPad Prism.

### NMR spectroscopy

Final NMR samples contained 0.1–2.0 mM DNA in 20 mM potassium phosphate, 80 mM KCl (pH 7.4), or in the low-salt solution, 2 mM potassium phosphate, 8 mM KCl, pH 7.4 buffer. All NMR experiments were performed on an Agilent 800 MHz spectrometer. Homonuclear 2D NMR experiments, including DQF-COSY, TOCSY, and NOESY, were collected at 298 K for the benzothiazole–*MYC* G4 complex in both water and D_2_O at 100 and 10 mM K^+^. NOESY mixing times were set from 50 to 300 ms, and the TOCSY mixing time was 70 ms. WATERGATE and excitation-sculpting were employed for water suppression. Spectra were processed using NMRPipe, with Sparky (UCSF) utilized cross-peak assignments and integrations.

### Structure calculation

The topology parameters of BTO-28 were generated from the Automated Topology Builder server (http://atb.uq.edu.au/) and integrated into XPLOR-NIH (version 2.47). Distances between nonexchangeable protons were derived from NOESY spectra and classified into four categories: strong (1.8–2.9 Å), medium (1.8–3.5 Å), weak (1.8–5.0 Å), and very weak (1.8–6.0 Å). The cross-peak between the thymine methyl and H6 protons (3.0 Å) served as a reference. Glycosidic torsion angles of 240 ± 70° for *anti* were applied based on intraresidue H8-H1′ NOE intensity. The planarity restraints were applied to the G-tetrads. The structural calculations of complex were carried out using a simulated annealing protocol as previously described [47], started from the coordinate of an extended DNA chain and two BTO-28 molecules. The model was first heated to 3000 K and kept for 10 ps. The van der Waals (vdW) forces were turned off in the heating stage. The system was subsequently cooled to 25 K within 24 ps using a combined energy function composed of geometric, vdW, and NOE terms. The structure was subjected to final energy minimization for 40 ps. Among the 200 structures calculated in the last iteration, 10 structures with the lowest energies and minimal restraint violations were selected for 3D representation.

### Differential scanning calorimetry

Differential scanning calorimetry (DSC) measurements were performed using an automated VP-CAP-DSC microcalorimeter (Malvern Inc., USA). G4 DNA at 100 μM was investigated both in the presence of BTO-28 at a 1:4 DNA-to-ligand molar ratio and in its absence. Temperature scans were performed from 25°C to 105°C at a rate of 1.0°C/min. A buffer–buffer scan was subtracted from each buffer–sample scan, and linear-polynomial baselines were applied to each dataset. The corrected thermograms were then normalized to the single-stranded DNA concentration to derive molar heat capacity curves. The melting temperature (*T*_m_) was determined as the peak temperature in each thermogram.

### *Taq* DNA polymerase stop assay

5′-d(TAATACGACTCACTATAGCAATTGCGTG)-3′ was 5′-end-labeled with FAM. The labeled primer was annealed to the DNA template (Supplementary Table S1) in 50 mM Tris (pH 7.4) using the previously described protocol [48]. The specified compounds were added to 20 nM annealed template in 50 mM Tris (pH 7.4), 1 mM MgCl_2_, 5 mM dithiothreitol (DTT), dNTP mixture, and 5 U/μl *Taq* polymerase (Takara, RR001A). Samples were incubated for 40 min at 64°C for polymerase extension. The reaction was stopped by adding alkaline gel loading dye, and the samples were analyzed on a 16% denaturing polyacrylamide gel.

### Cell lines

The HT-29 cells were cultured in McCoy’s 5A (Gibco, 16600082) with 10% fetal bovine serum (Gibco, A5669701), 100 U/ml penicillin and 100 mg/ml streptomycin. The 293T cells were cultured in DMEM (Gibco, 11965092) with 10% fetal bovine serum, 100 U/ml penicillin and 100 mg/ml streptomycin. HK-2 cells were cultured in K-SFM (Invitrogen, 17005042) supplemented with Gentamicin/Amphotericin solution (Gibco, R01510). Cells were cultured in a Thermo Fisher Scientific (Waltham, MA) water-jacketed incubator at 37 °C with 5% CO_2_.

### Cell viability assays

Cell viability assay was estimated using the CellTiter blue (Promega, G8081). Cells were seeded in triplicate at a density of 8000 cells per well onto 96-well plates containing 100 μl of medium per well and incubated for 12 h. The drug, diluted in complete medium, was then added to each well. Cell viability was assessed by measuring absorbance at 590 nm using an EnVision microplate reader (PerkinElmer).

### Quantitative real-time PCR

Total mRNA was isolated with a MiniBEST Universal RNA Extraction Kit (TaKaRa, 9767), and complementary DNA synthesis was performed with a PrimeScriptRT Mater Mix (TaKaRa, RR036A), per the manufacturers’ instructions. Quantitative polymerase chain reaction (qPCR) analysis was conducted on a QuantStudio 6 Flex Real-Time PCR (Applied Biosystems) with TB Green Premix Ex Taq II FAST qPCR (TaKaRa, CN830S). Expression levels were quantified with the ΔΔ*C*_t_ method and normalized to β-actin mRNA. Sequences of primers used are listed in Supplementary Table S1.

### Chromatin immunoprecipitation qPCR

Approximately 1 × 10^8^ HT-29 cells were treated with DMSO or BTO-28 (1000 nM) and cross-linked with 1% formaldehyde at 37°C for 10 min. Chromatin immunoprecipitation (ChIP) assays were performed using SimpleChIP Enzymatic Chromatin IP Kit (Cell Signaling Technology, 9003S), according to the manufacturer’s instructions. Chromatin was immunoprecipitated overnight at 4°C with 2 μg of the antibody of interest. Antibody-bound complexes were subsequently isolated using ChIP-Grade Protein G Magnetic Beads (Cell Signaling Technology, 9006). Immunoprecipitated DNA was analyzed using qPCR on a QuantStudio 6 Flex Real-Time PCR (Applied Biosystems) with TB Green Premix Ex Taq II FAST qPCR (TaKaRa, CN830S) and calculated as % of input. The antibodies against the SP1 (Abcam, ab231778) and RNA polymerase II CTD repeat YSPTSPS (Abcam, ab26721) were used for ChIP assays. All antibodies used were certified as ChIP-grade by the manufacturer. Normal rabbit IgG (Cell Signaling Technology, 2729) was used as a background control. Details of the sequences of the primers are provided in Supplementary Table S1.

### Dual-luciferase assays

The MYC promoter containing the promoter elements P1 (including NHE III_1_) and P2 was cloned into the pGL3 vector (Promega, Madison, WI) upstream of the firefly luciferase reporter gene. The G4-forming sequence and its variants located upstream of the luciferase reporter gene in the plasmids used in this study are listed in Supplementary Table S1. All fragments inserted into the pGL3 basic vectors were synthesized by Tsingke. The plasmids were purified using the TaKaRa MiniBEST Endo-free Plasmid Purification Kit (Takara, 9783S) and then transfected into 293T cells along with the pRL-TK control reporter vector. Cells were incubated with BTO-28 for 24 h. Relative luciferase activities were measured using the Dual-Luciferase Reporter Assay System (Promega, E1910). Each group was conducted in triplicate.

## Results

### BTO-28 binds to *MYC* G4 with high affinity

Quinoline moiety has been shown to stabilize G4 conformations [49, 50], and introduction of positive charges into aliphatic side chain modulates the G4 binding affinity by interfering electrostatic interactions [51]. Our previous studies demonstrated that within the 1-methylquinolinium unit, modification at 2-position could induce the selectivity toward double-stranded DNA (dsDNA) or non-B DNA [52], but the underlying mechanisms governing these distinct recognition abilities remain unclear. In this work, we developed a benzothiazole derivative, BTO-28, which is linked to a 1-methylquinolinium unit, and the functionalization of the molecule with an extra methyl group at 2-position and a cationic side chain aims to modulate its interactions with G4 structures.

Using 1D ^1^H NMR spectroscopy, we investigated the interaction between BTO-28 and the *MYC* G4 under 10 mM K^+^ conditions. Upon titration of BTO-28 at low concentrations (ratios from 0 to 1), imino protons of *MYC* G4 were perturbed and a separate set of imino signals emerged (Fig. 1D), which indicates binding interactions. The original imino proton peaks of the free *MYC* G4 remained visible, suggesting that BTO-28 binds to *MYC* G4 in a slow-exchange manner on the NMR chemical shift time scale, indicative of high-affinity interactions [53]. Further addition of the molecule induced the appearance of 12 distinct imino proton signals with sharp spectra line widths, indicating the major conformation of BTO-28–*MYC* G4 complex in solution. Significant perturbations were detected at G15 (in 3′ G-tetrad) and G8 (in 5′ G-tetrad), while the imino protons of the central G-tetrad exhibited minimal chemical shift changes (Supplementary Fig. S1). These perturbations saturated at concentrations exceeding four molar equivalents of BTO-28. Under 100 mM K^+^ conditions, the NMR titration spectra exhibited a similar binding behavior (Supplementary Fig. S2).

We also visualized BTO-28–bound *MYC* G4 using mass spectrometry [54, 55]. Mass spectrometry analysis at increasing ratios of BTO-28 to *MYC* G4 revealed that the 2:1 complex dominates and that a third binding site is not observed (Supplementary Fig. S3). These findings indicate that BTO-28 interacts mainly with two high-affinity binding sites in *MYC* G4, resulting in a 2:1 stoichiometry. The CD spectra retained characteristics of the parallel folding of *MYC* G4 [56], evidenced by a positive band near 260 nm and a negative band at 240 nm (Supplementary Fig. S4). BTO-28 interacts with high-affinity site of *MYC* G4 with a *K*_D_ value of 6.8 nM, demonstrating stronger binding than observed for other G4s or dsDNA (Supplementary Fig. S5). These results indicate that BTO-28 binds effectively to the *MYC* G4, with a higher performance compared to other G4 binding compounds [46, 47, 57].

### Ligand binding stabilizes G4 structure

**Figure 2.**
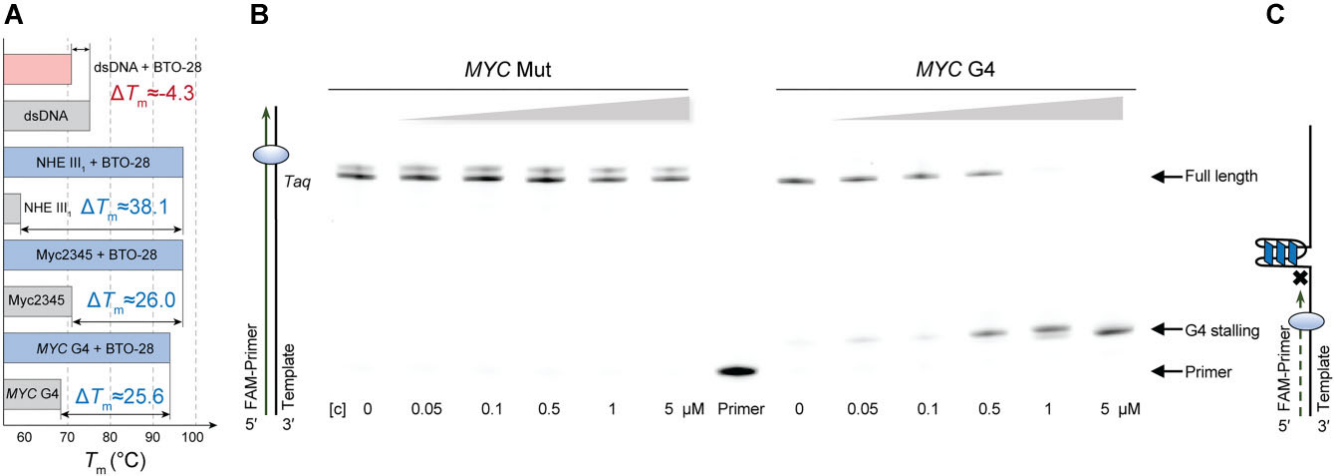
Ligand binding stabilizes *MYC* G4. (**A**) DSC melting temperatures of *MYC* G4, Myc2345, NHE III_1_, and dsDNA with or without BTO-28. (**B**) *Taq* polymerase stop assays with increasing concentrations of BTO-28 (0.05, 0.1, 0.5, 1, and 5 μM). The right six panels show the scan for *MYC* G4, while the left six panels show the scan for *MYC* G4-mutant. (**C**) Schematic representation of the polymerase stop assay. The FAM-labeled primer (dark green) is annealed to a G4-forming DNA template (black) and extended by *Taq* polymerase (light blue).

Further biophysical analyses were conducted to investigate BTO-28 interaction with *MYC* G4. Differential scanning calorimetry demonstrated that binding with BTO-28 markedly increased the thermal stability of the G-quadruplex. Specifically, the melting temperatures of *MYC* G4 rose by 25.6°C for *MYC* G4 and that of Myc2345 by 26.0°C (Fig. 2A and Supplementary Fig. S6). BTO-28 also significantly enhanced the stability of NHE III_1_. In contrast, the molecule destabilized dsDNA, lowering its melting temperature by 4.3°C, likely accounting for its much lower binding affinity toward dsDNA.

The contribution of the high affinity of BTO-28 to G4 stabilization was further investigated by DNA polymerase stop assay. The embedded *MYC* G4 sequence in the template could form into G4 structure that arrests the polymerase processivity [48, 58]. The mutated sequence that lacks G4-forming ability served as the negative control (Supplementary Table S1). BTO-28 significantly accumulated the truncated products in a concentration-dependent manner, and the full-length extension products became barely appreciable at high BTO-28 concentrations (Fig. 2B). Primer extension using mutated *MYC* templates indicated that no significant stalling occurred in the presence of the compound (Fig. 2B). These results support that BTO-28 induces G4 formation in the G-rich templates, which impedes DNA synthesis (Fig. 2C).

### Comprehensive proton assignment of the 2:1 benzothiazole–*MYC* G4 complex

**Figure 3.**
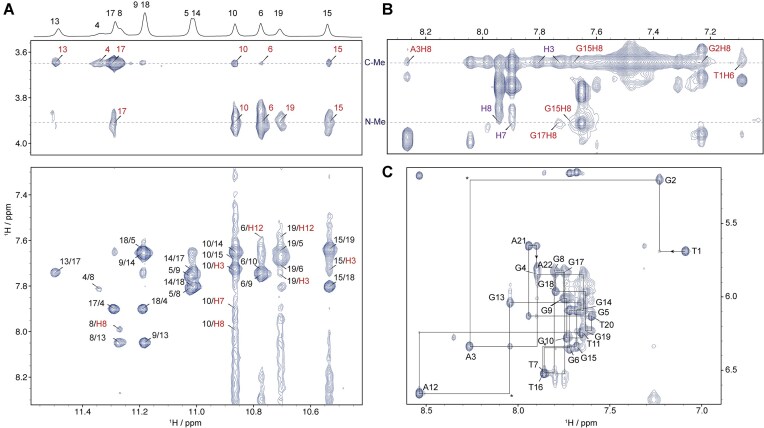
2D ^1^H-^1^H NOESY spectra of the 2:1 BTO-28–*MYC* G4 complex. (**A**) Intermolecular NOE cross-peaks (in red) between BTO-28 and *MYC* G4 imino protons. (**B**) Intermolecular NOE contacts between BTO-28 and *MYC* G4 H6/H8 aromatic protons. BTO-28 protons are labeled in red, and intramolecular BTO-28 cross-peaks are labeled in purple. (**C**) Sequential H6–H1′ and H8–H1′ connections (solid lines). Asterisks indicate missing cross-peaks. All spectra were acquired at 298K in 10 mM K^+^ (pH 7.4) with a 300 ms NOESY mixing time.

Oligonucleotides with residue-specifically ^15^N-labeled guanines were prepared and then complexed with the molecule. The imino H1 protons were assigned (Supplementary Fig. S7A) by ^1^H-^15^N HSQC experiments [59] and were further validated by inter-residue NOE correlations. A series of 2D NMR spectra were performed (Fig. 3 and Supplementary Figs S8–S10) to completely assign the nonexchangeable protons and intermolecular NOEs of BTO-28–*MYC* G4 complex. Each proton showed a single chemical shift (Supplementary Table S2), indicating that BTO-28 binds to the *MYC* G4 in a single dominant conformation. The characteristic H1–H8 NOEs in G-tetrads combined with the sequential inter-residue H1′-H6/H8 NOE connectivities defined the *MYC* G4 scaffold (Fig. 3C). Under low ionic strength of 10 mM K^+^, the BTO-28–*MYC* G4 complex displayed sharper cross-peaks, suggesting that the stronger ligand binding retards the exchange with possible minor conformations. A single set of proton peaks was observed for the 5′ and 3′-end overhang residues, suggesting a well-defined stacked conformation at both ends (Supplementary Tables S3 and S4), in contrast to the complicated patterns observed at 100 mM K⁺ (Supplementary Fig. S11). Same complex structures were formed under both conditions, as indicated by the nearly identical resolved regions of the NMR spectra.

From the NOESY spectra, intermolecular NOEs between BTO-28 and *MYC* G4 protons were identified, and protons of BTO-28 in the bound state were clearly assigned (Supplementary Tables S5–S7). Two sets of methyl signals from BTO-28, labeled C-Me and N-Me, appear at ∼3.6 and 3.9 ppm, respectively (Fig. 3A and B). Because these signals are located in a region free from overlapping DNA resonances, multiple intermolecular cross-peaks with tetrad H1 imino protons were clearly assigned. Comparisons of proton chemical shifts between the free and bound states reveal substantial conformational rearrangements in both the 5′- and 3′-flanking regions (Supplementary Fig. S1). Loop residues T7 and T16 show only minor chemical shift changes, suggesting they are not part of the primary binding interface.

### NMR structure establishes ligand binding mode

**Figure 4.**
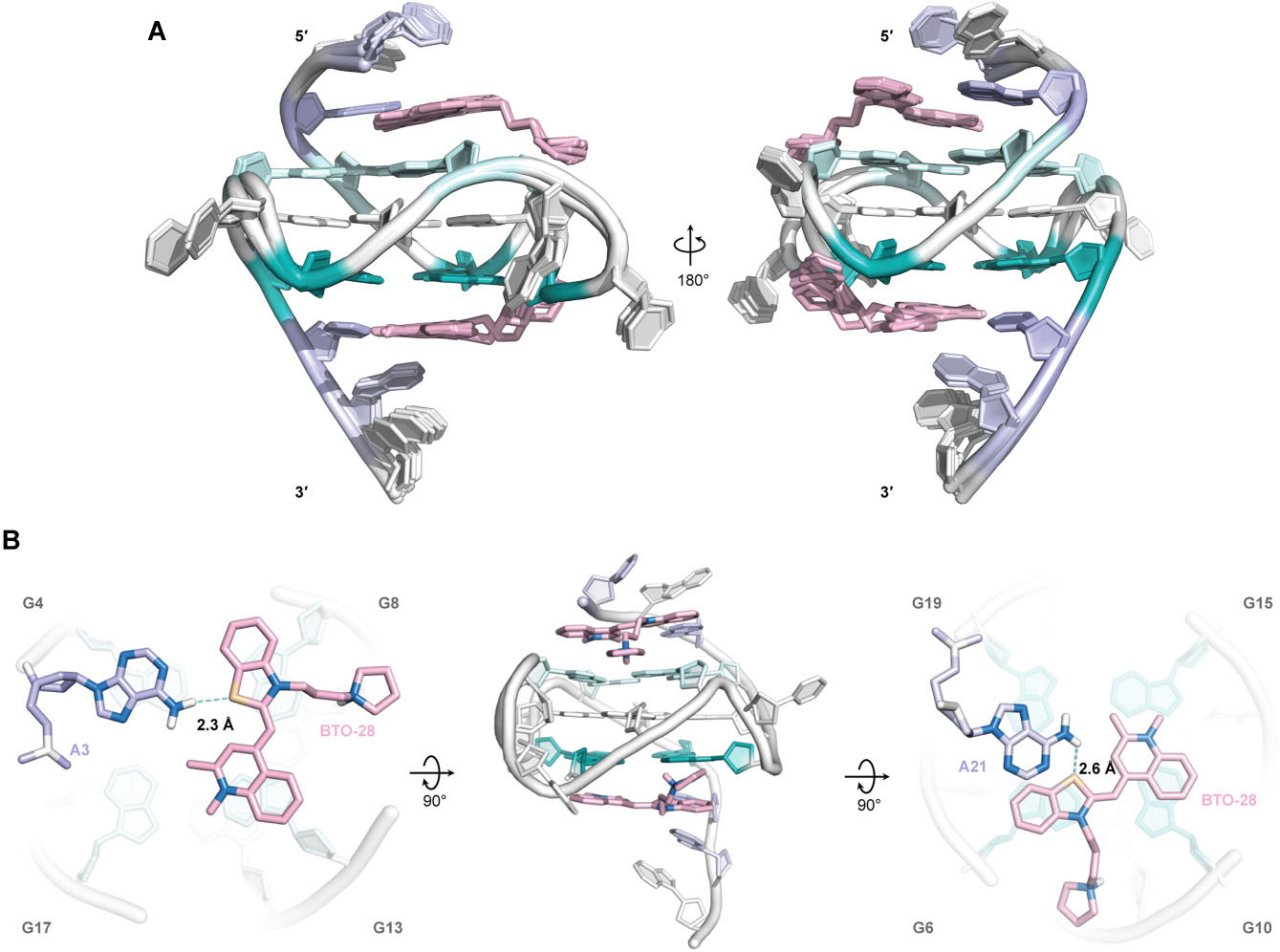
BTO-28–*MYC* G4 complex reveals additional stacked layers at both termini and flanking-residue rearrangements. (**A**) Superposition of 10 lowest-energy structures and a representative structure (**B**) of the 2:1 BTO-28–*MYC* G4 complex (PDB: 9L4E), shown from the top (left), side (middle), and bottom (right) perspectives. The 5′-tetrad residues are highlighted in pale cyan; the 3′-tetrad residues are in teal; and the flanking residues are in light blue. BTO-28 molecules are depicted in light pink.

**Figure 5.**
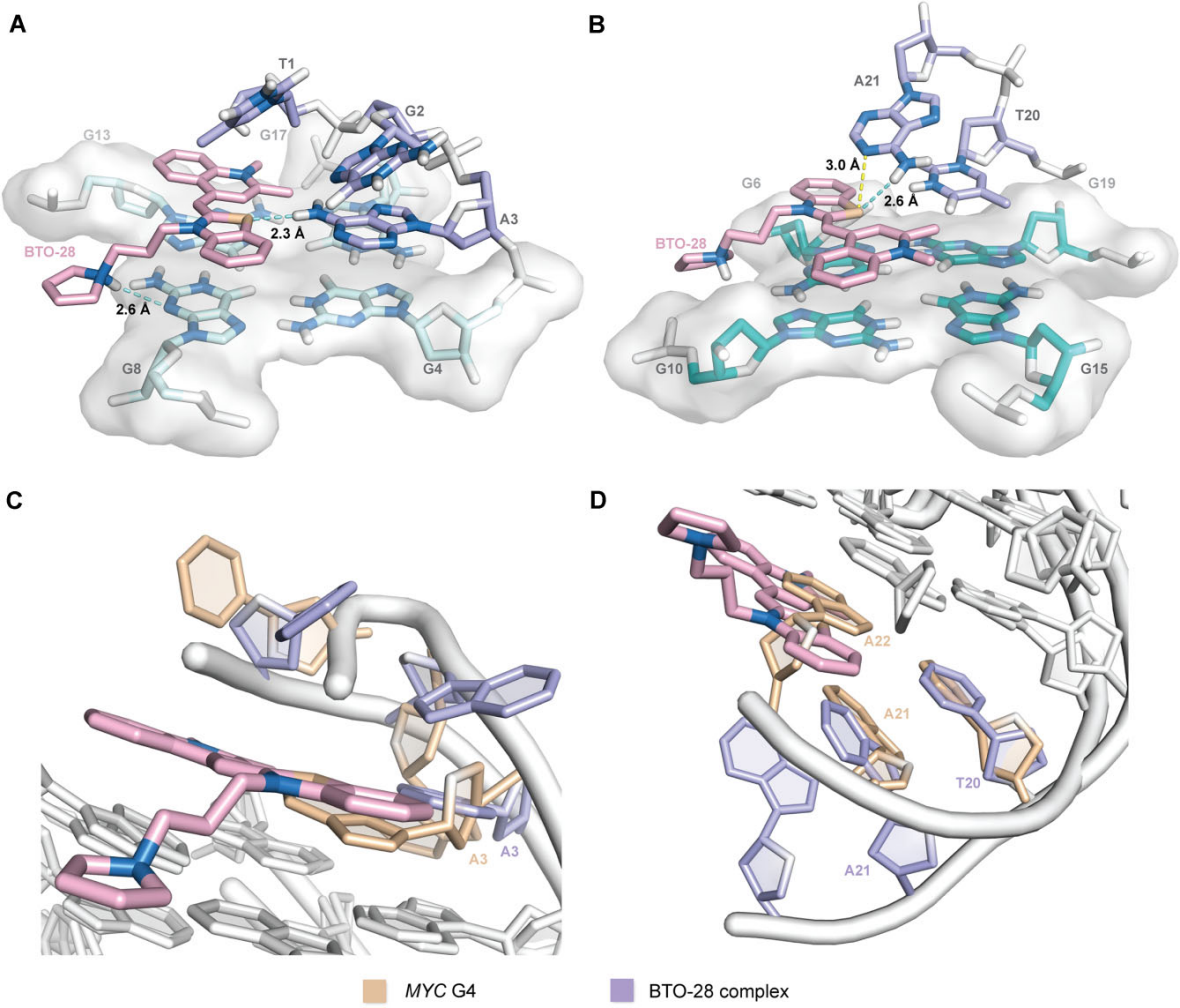
BTO-28 binding explains *MYC* G4 recognition. (A, B) Expanded illustrations of the BTO-28–*MYC* G4 complex at the 5′ (**A**) and 3′ (**B**) ends. Hydrogen bonds are shown as cyan dashed lines, and chalcogen bonds are shown as yellow dashed lines. (C, D) Structural comparisons illustrate conformational differences between the free (PDB: 1XAV) and BTO-28–bound *MYC* G4 at the 5′ (**C**) and 3′ (**D**) ends. Superimposed structures highlight reorientation of the flanking residues, each color-coded as indicated.

Based on NOE-derived intra- and inter-residue distance restraints, along with glycosidic torsion angle and G-tetrad planarity restraints, we employed restrained molecular dynamics to determine the high-resolution structure of the BTO-28–*MYC* G4 complex. The assembly of the 10 lowest-energy structures showed high convergence, with all heavy atoms root-mean-square deviation (r.m.s.d.) of 0.4 and 0.13 Å for all residues and G-core, respectively (Fig. 4A, PDB ID 9L4E; Table 1). The BTO-28–bound *MYC* G4 maintains its parallel-stranded topology with three propeller loops, yet the flanking regions at both ends rearrange significantly. BTO-28 engages both the 5′- and 3′-end of the *MYC* G4. The benzothiazole and quinoline rings engage in π–π stacking with the G-tetrads, while its pyrrolidine side chain is accommodated within the groove (Fig. 4B). Compared to the free form, the residue A3 at 5′-end reorients so that its amino group now points toward the central axis (Fig. 5C), as indicated by NOE contacts of A3H2 to G4H1′ and BTO-28 H12/H13 protons. This conformation allows the benzothiazole sulfur atom to be involved in hydrogen bonding interaction with the A3 amino group. Earlier investigations on benzoselenazole compounds suggested that this type of interaction is possible [57]. The well-stacked position of A3 was also supported by the profound downfield shifting of its H2 and H8 protons and an upfield shifting in G4H1 (Supplementary Fig. S1). Meanwhile, T1 and G2 reposition to stack over the BTO-28–adenine plane. Under these conditions, BTO-28 adopts a more planar conformation that aligns with the external tetrad, covering G8 and G13 (Fig. 5A). Intermolecular NOEs connecting G8 imino protons to the BTO-28 benzothiazole moiety and G13 imino protons to the BTO-28 quinoline moiety indicate that BTO-28 is anchored in a defined conformation (Supplementary Fig. S12A). Additionally, intermolecular NOEs involving both BTO-28 methyl groups and G17H1 protons were also observed, and intramolecular NOEs involving BTO-28 H3/H12 and H5/H9 protons provided supplementary validation of these ligand-induced conformational states. The presence of adjacent bases in the binding pocket might stabilize the coplanar immobilization of the benzothiazole and quinolinium groups [60–62], consistent with the fluorescence enhancement upon binding to G-quadruplex [63].

**Table 1.** Structural and NMR statistics for the 2:1 BTO-28–MYC G4 complex

Structure statistics	
NOE-based distance restraints	
Intraresidue	579
Inter-residue	271
Sequential	184
Long range	30
Medium range	57
BTO-28–G4	102
Deviations from idealized geometry	
Bonds (Å)	0.004 ± 0.000
Angles (°)	0.596 ± 0.004
Impropers (°)	0.364 ± 0.041
NOE violations	
r.m.s.d. of violations	0.043 ± 0.001
Pairwise r.m.s.d. of heavy atoms (Å)	
G-tetrad core	0.13 ± 0.06
All residues	0.45 ± 0.14

At the 3′-end, the initial stacking mode of A21 over the T20-A22 base pairing is disrupted, and instead, T20 integrates into the BTO-28-base plane stacking on the 3′-tetrad (Fig. 5B). NOE connections linking A21H2 to the benzothiazole aromatic protons further confirm the new capping conformation of the 3′-overhang residues, which ultimately forms a new binding pocket (Fig. 5D). This arrangement enables a hydrogen bond between the A21 NH_2_ group and the BTO-28 benzothiazole sulfur atom. Numerous NOE cross-peaks between the BTO-28 aromatic protons and G6H1/H8 as well as G10H1/H8 revealed clearly defined orientations of the benzothiazole molecule (Supplementary Fig. S12B). Two methyl groups within the quinoline unit point to the emptier region adjacent to the groove bordered by G15 to G19. This is supported by medium-intensity NOE cross-peaks involving all the imino protons of the 3′-tetrad and the N-Me group. Alternatively, the 3′-terminal A22 conformations remain flexible, exhibiting few NOE interactions.

The side chain of BTO-28 plays a considerable role in facilitating specific interactions with *MYC* G4. Although the protonated HN3 could not be observed due to rapid solvent exchange, intermolecular NOE connections between the benzothiazole H6′ protons and G8H21/H1′ demonstrate that the pyrrolidine side chain accommodates into the groove bordered by G8 to G13. Additionally, a potential hydrogen bond is formed between G8N3 and HN3 with a distance of ∼2.6 Å (Fig. 5A), with the N3 nitrogen of guanine as the acceptor [64]. Lower salt concentration might strengthen this hydrogen bond [65], which may account for the higher benzothiazole binding observed at the 5′ end. At the 3′-terminal, similar NOE pattern was observed between the BTO-28 side chain and G6. However, the protonated HN3 does not exhibit a close spatial proximity to G6N3, suggesting a weaker yet plausible hydrogen bonding interaction. The flexibility of residue T7 along with the hydrogen bond between A21 and benzothiazole likely limits the ligand–groove interaction. This effect may also explain the stronger affinity observed for the *MYC* G4 at 5′ end. Collectively, NMR structure establishes a binding mode in which one BTO-28 molecule covers the top G-tetrad, while another covers the bottom G-tetrad.

We also conducted 1D ^1^H NMR titration and ^1^H-^15^N-HSQC experiments of BTO-28 with the Myc2345 sequence [40]. Myc2345 contains the conserved guanine at position 20 in the 3′-flanking sequence, where *MYC* G4 includes a G-to-T mutation to reduce the conformational polymorphism [37]. The resemblance of the imino proton resonances in the 2:1 BTO-28 complexed with *MYC* G4 and Myc2345 indicates that both sequences share a similar G-quadruplex topology (Supplementary Fig. S7), which is consistent with the same binding pattern observed in mass spectrometry (Supplementary Figs S3 and S13).

### BTO-28 regulates *MYC* transcription

**Figure 6.**
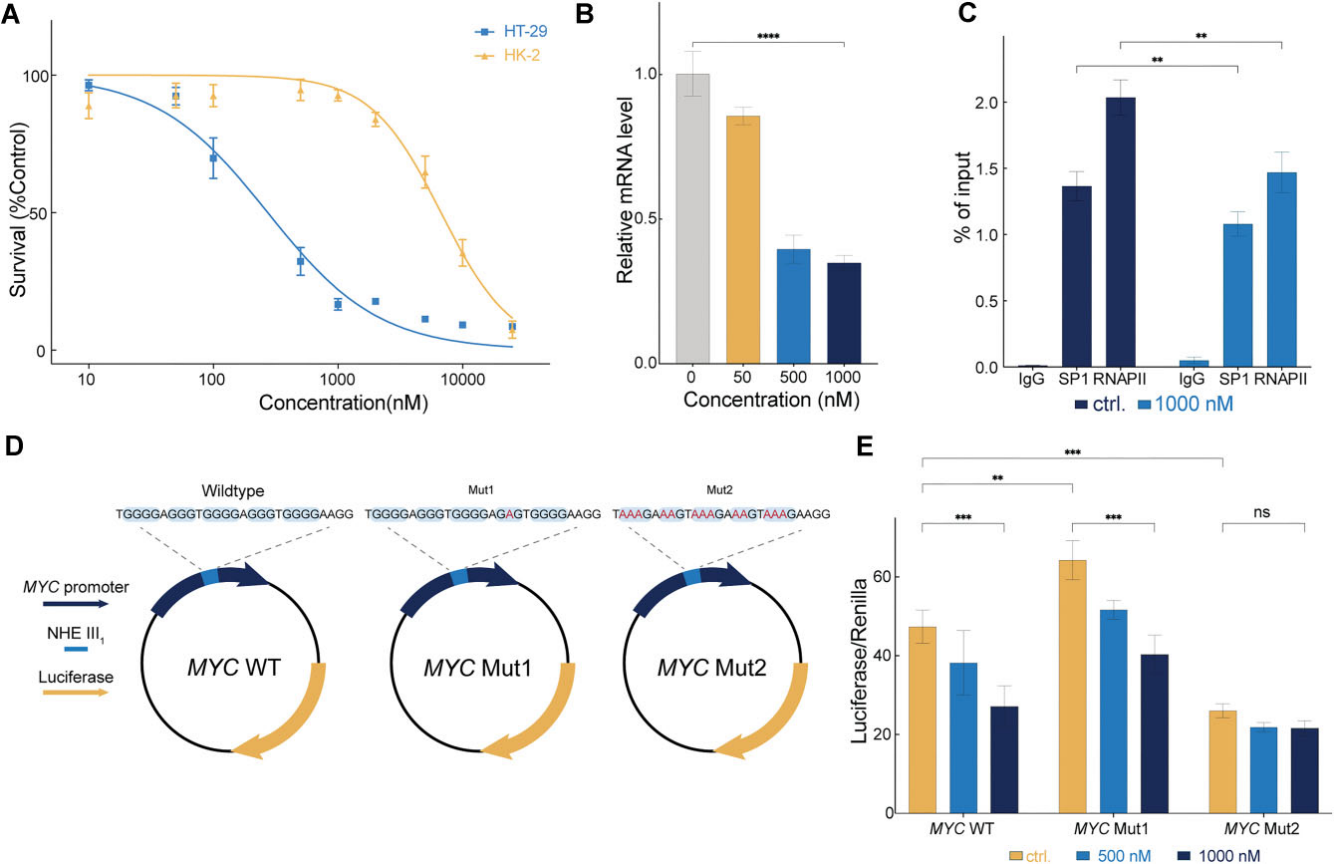
BTO-28 regulates *MYC* transcription. (**A**) Effects of compounds on the proliferation of different cell lines. Data represent the mean of three independent experiments. (**B**) Quantification of *MYC* mRNA in HT-29 cells treated with the quantified BTO-28 compounds. All mRNA levels are normalized to β-actin, and the ΔΔ*C*_t_ method was employed for relative gene expression analysis. Error bars represent the standard deviation from three biological replicates. ****P < .0001. (**C**) ChIP of the interaction between SP1 and the *MYC* promoter shows that BTO-28 disrupts SP1 binding to the G4-forming region of the *MYC* promoter. *n* = 4 biologically independent samples. Error bars represent the mean ± standard deviation (SD). Statistical significance was determined using Bonferroni’s multiple comparisons test following a two-way ANOVA. **P < .01. (**D**) Schematic diagram of plasmids containing NHE III_1_ region and its variants cloned upstream of the luciferase reporter gene. (**E**) Relative luciferase expression by the *MYC* promoters normalized to the renilla plasmid after treatment with BTO-28. Error bars represent the mean ± SD (*n* = 3). *t* test. ns, nonsignificant difference; **P < .01; ***P < .001.

Cell viability assays revealed that BTO-28 effectively inhibited the proliferation of colorectal carcinoma (HT-29) cells, with IC_50_ values of ∼273 nM, indicating a higher level of activity compared to other G4 ligands [39, 66]. Notably, it did not inhibit the growth of normal kidney cells (HK-2) at concentrations up to 5 μM (Fig. 6A). Given that proto-oncogenes such as *MYC*, *KRAS*, *RET*, and *VEGF* are often overexpressed in human cancers, we analyzed the regulatory effect of BTO-28 on proto-oncogene transcription in HT-29 cells. *MYC* downregulation mediated by G4 stabilization was measured using P1-promoter primers [28, 30]. Downregulation of P1-driven MYC expression was observed with BTO-28 (Fig. 6B). In contrast, treatment with BTO-28 did not cause a concentration-dependent decrease in the expression of *RET*, *hTERT*, *KIT*, *KRAS*, and *VEGF* in HT-29 cells compared to controls (Supplementary Fig. S14).

To determine whether the reduced MYC transcription caused by BTO-28 is related to the inhibition of SP1 binding to the *MYC* promoter, we performed ChIP analysis on HT-29 cells. Treatment with BTO-28 resulted in a decrease in SP1 occupancy at the *MYC* promoter (Fig. 6C). The formation of G4-DNA structures may impede the transcription elongation by RNA polymerase II (RNAPII), thereby inhibiting gene expression [67]. Aligned with this, cells treated with BTO-28 showed reductions in RNAPII binding at the *MYC* promoter. Collectively, these results indicate that BTO-28 may disrupt the interaction between these transcription factors and the *MYC* promoter G4.

Dual-luciferase assay has been used to examine the effect of G4 formation in NHE III_1_ on gene transcription. The *MYC* promoter that contains NHE III_1_ and the two major promoter regions, P1 and P2, was cloned into the pGL3 vector upstream of the firefly luciferase reporter gene to obtain the construct *MYC* WT [68]. An earlier study demonstrated that the *MYC* Mut1 construct, which contains a single G-to-A substitution that destabilizes the G4 motif, resulted in a significant increase in transcriptional activation compared to that of the wild-type NHE III_1_ sequence [6]. The primary G-quadruplex structure within the NHE III_1_ region responsible for *MYC* transcription silencing involves four consecutive G-runs at the 3′-end [48]. A second mutant in which all core guanines involved in G4 scaffold were replaced by adenines was constructed to completely obstruct G4 formation (Fig. 6D). Compared to the wild-type promoter, introducing a single-base mutation enhanced promoter activity in cells. While the promoter activity of *MYC* Mut2, which contains multiple point mutations sufficient to prevent G-quadruplex formation, was diminished (Fig. 6E), this reduction suggests that NHE III_1_ with core guanine substitutions may impede transcription complexes or inhibit the binding of positive transcription factors, leading to decreased transcription levels [69].

BTO-28 significantly reduced the luciferase activity of the reporter construct containing wild-type *MYC* compared to the control. Although the single-base mutant exhibited enhanced promoter activity compared to the wild type, modest reductions were observed at high drug concentrations. This suggests that the stabilizing effects of BTO-28 can partially counteract the destabilizing impact of the single-base mutation. Intriguingly, BTO-28 did not inhibit the luciferase expression of *MYC* Mut2 (Fig. 6E). These results suggest that BTO-28 likely inhibits *MYC* transcription by influencing specific G-quadruplex structure within the NHE III_1_ region.

## Discussion

Comprehensive NMR structural studies of BTO-28 in complex with *MYC* G4 offer precise insights into the molecular interactions driving G-quadruplex recognition. Systematic structural analyses of various ligand complexes uncovered a shared binding mode: small, arc-shaped molecules feature a broad planar surface for π–π stacking on the external G-tetrads [37, 40]. They also engage flexible flanking residues at both 5′ and 3′ ends, adapting their conformation to maintain stable binding [70]. Hydrogen bonding involving the recruited residue is scarce in previously characterized complexes and has only been observed at the 3′-end for quindoline [65] and DC-34 [71] bound to the *MYC* G4. Here, for the first time, we describe a unique hydrogen-bonding pattern between a nucleobase surrogate and the flanking bases. The sulfur in the benzothiazole core forms hydrogen bonds with the NH_2_ groups of A3 and A21, respectively, and engages in chalcogen bonds with adenine N1 atom. BTO-28 might interfere with canonical base pairing in dsDNA, leading to decreased stability. Conversely, upon binding to *MYC* G4, the molecule utilizes a base recruitment mechanism to reinforce the structure through hydrogen bonding and stacking interactions. Through these interactions with the G4, BTO-28 establishes a distinct binding mode not observed for previously reported ligands.

Incorporating positive charges into attached side chains is a commonly used strategy for promoting G4 binding, primarily by leveraging electrostatic interactions [51]. In quindoline derivatives, for instance, the diethylamino group resides 2.7 Å from the DNA phosphate backbone, whereas the azepane ring in DC-34 is too short and bulky to support such an interaction (Supplementary Fig. S15) [71]. By contrast, the optimal number of carbon atoms linking the benzothiazole core to the amine in BTO-28, combined with the appropriate steric hindrance of the flexible pyrrolidine, facilitates hydrogen bonding with the 5′ external G-tetrad and electrostatic engagement within G4 grooves. Such interactions appear to stabilize and properly orient the benzothiazole, which may underlie the observed comparative specificity in chemical and biological assays. No interaction has been documented previously between positively charged side chains and the external G-tetrads, highlighting the importance of refining ligand–G4 interface within the groove dimensions. Changing the pyrrolidine ring to other aliphatic amines led to small decreases in potency, whereas attempts with morpholine heterocycles yielded uniformly inferior results. In parallel, altering the 2-position methyl on 1-methylquinolinium to styryl moieties significantly reduced activity (Supplementary Fig. S16). Subtle adjustments to the shape and electrostatic properties of the ligand will influence its precise orientation within the intercalated pockets. Benzothiazole binding at the 5′-flanking region is driven strongly by specific residue interactions, whereas 3′-end binding appears more dependent on the surrounding ionic environment. These variations reflect inherent structural differences: the 5′-terminal is more hydrophobic, promoting reduced solvent exposure and strengthened hydrogen bonds [72], while the 3′-terminal remains relatively hydrophilic and less favorable for ligand stacking. This contrast explains the stronger binding affinity measured at the 5′ end. It is also worth noting that the wild-type Myc2345 sequence appears to adopt the same conformation in the 2:1 benzothiazole complex.

Our research targeted the recognition of the G4 element within the human *MYC* NHE III_1_ locus. Multiple independent assays demonstrated that BTO-28 stabilizes the G4 structure and promotes the refolding of DNA flanking regions. These results present a potentially mechanism of *MYC* transcriptional inhibition via G4 targeting. While BTO-28 shows strong binding to *MYC* G4, the widespread presence of G4 motifs across the genome may lead to the risk of off-target effects by engaging unrelated G4 structures in other mammalian genes. We envision that integrating BTO-28 with recently developed ATENA (Approach to Target Exact Nucleic Acid alternative structures) will enable precise targeting for *MYC* G4 [30], thereby minimizing unintended interactions with non-relevant G4s.

## Conclusions

Overall, our study of the interaction between *MYC* G-quadruplex and BTO-28 provides valuable insights into how ligand-induced structural changes in G4 DNA may orchestrate complex transcriptional processes. BTO-28 exhibits unique binding at the 5′- and 3′-termini of the *MYC* G4. By uncovering base sequences critical for low nanomolar affinity, we highlight that stacking interactions and groove interactions together enable G4 targeting. Also, our study indicates that G-quadruplex formation is intricately linked to *MYC* gene expression. These findings provide an important rationale for end-specific interactions of BTO-28 in shaping *MYC* G4 stability and functionality.

## Supplementary Material

gkaf888_Supplemental_File

## Data Availability

The coordinates for structures of the 2:1 BTO-28–MYC G4 complex (PDB code: 9L4E) have been deposited in the Protein Data Bank. Chemical shift assignments were also deposited in BMRB with accession code 36722.
