# Supplementary material for: Molecular recognition and effects of a benzothiazole derivative targeting the MYC G-quadruplex
Source: Nucleic Acids Res. 2025 Sep 12;53(17):gkaf888. doi: 10.1093/nar/gkaf888 (PMC12445692; doi:10.1093/nar/gkaf888)
Supplement: gkaf888_Supplemental_File [file gkaf888_supplemental_file.pdf]

Supplementary Information

Molecular Recognition and Effects of a Benzothiazole Derivative Targeting the *MYC* G-quadruplex

Xiao Ni[1], Xiaodong Hu[1], Wei Long[2], Wenxian Lan[3], Chunxi Wang[1], Wing-Leung Wong[2,*] and Chunyang Cao[1,*]

[1]State Key Laboratory of Chemical Biology, Shanghai Institute of Organic Chemistry, University of Chinese Academy of Sciences, Chinese Academy of Sciences, 345 Lingling Road, Shanghai 200032, China

[2]State Key Laboratory of Chemical Biology and Drug Discovery, Department of Applied Biology and Chemical Technology, The Hong Kong Polytechnic University, Hung Hom, Hong Kong SAR 999077, China

[3]The Core Facility Center of CAS Center for Excellence in Molecular Plant Sciences, Institute of Plant Physiology and Ecology, Chinese Academy of Sciences, 300 Fenglin Road, Shanghai 200032, China

*Correspondence to wing.leung.wong@polyu.edu.hk and ccao@mail.sioc.ac.cn

Table of Contents

1. Supplementary Figures 3

Figure S1. The chemical shift differences between free and bound *MYC* G4 3

Figure S2. 1D NMR titration spectra of BTO-28 with *MYC* G4 in 100 mM K^+. 3

Figure S3. Negative ESI mass spectra of *MYC* G4 with BTO-28 4

Figure S4. CD Spectrum of *MYC* G4 with BTO-28. 5

Figure S5. Binding curves of BTO-28 with different DNA sequences 5

Figure S6. DSC melting temperatures of BTO-28 with different DNA sequences 6

Figure S7. Imino proton assignments of BTO-28–*MYC* G4 and Myc2345 7

Figure S8. TOCSY spectrum of BTO-28–*MYC* G4 complex. 8

Figure S9. The expanded H1–H1 region of the NOESY spectrum 9

Figure S10. DQF-COSY spectrum of benzothiazole–*MYC* G4 complex 10

Figure S11. H8/H6–H1′ region of the 2D NOESY spectrum in 100 mM K^+ 11

Figure S12. Representative inter-molecular interactions within the complex 12

Figure S13. Negative ESI mass spectra of Myc2345 with BTO-28 13

Figure S14. qTR-PCR analysis of G4-associated genes treated by BTO-28 14

Figure S15. Structural comparison of *MYC* G4 complexes with different ligands 15

Figure S16. Cell viability assays of BTO-28 analogs 16

2. Supplementary tables 17

Table S1. Oligonucleotides used in this study 17

Table S2. ^{1}H chemical shifts of the 2:1 BTO-28-*MYC* G4 complex in 10 mM K^+. 19

Table S3. Intramolecular NOE interactions involving the 5′-terminal residues T1-G4 20

Table S4. Intramolecular NOE interactions involving the 3′-terminal residues G19-A23 21

Table S5. ^{1}H chemical shifts of BTO-28 in the 2:1 BTO-28-*MYC* G4 complex 22

Table S6. Intermolecular NOE cross-peaks between *MYC* G4 5′-end and BTO-28 23

Table S7. Intermolecular NOE cross-peaks between *MYC* G4 3′-end and BTO-28 23

3. Synthesis and characterization 24

Scheme S1. Synthetic path of BTO-28 24

Figure S17. ^{1}H NMR spectrum of BTO-28 24

Figure S18. High resolution mass spectrometry of BTO-28 24

1. Supplementary Figures

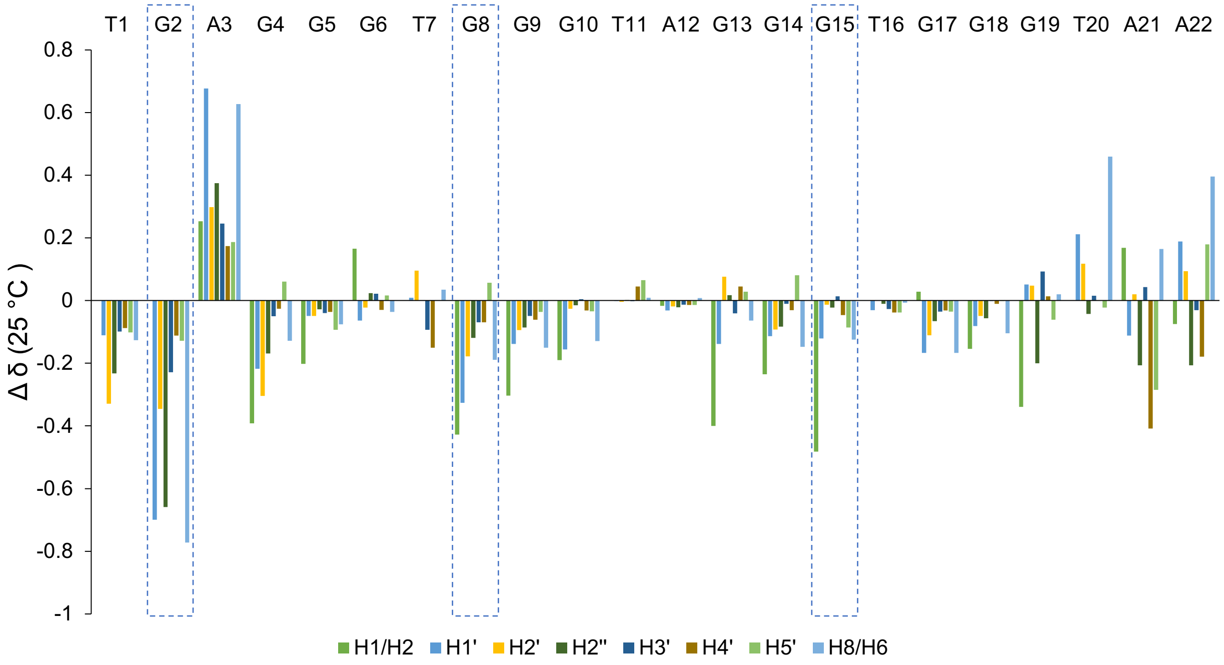

Figure S1. The chemical shift differences between free and bound *MYC* G4 at 25 °C. Residue numbering for *MYC* G4 follows the scheme presented in Figure 1A.

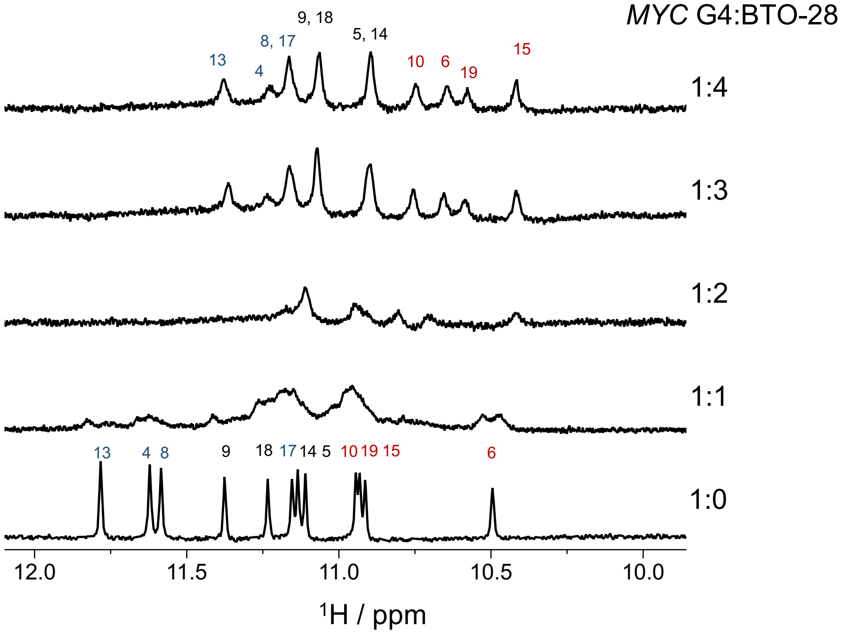

Figure S2. 1D ^{1}H NMR spectra of the imino proton region during BTO-28 titration into *MYC* G4 in 100 mM K^+, pH 7.4. Ratios of BTO-28 to *MYC* G4 are indicated.

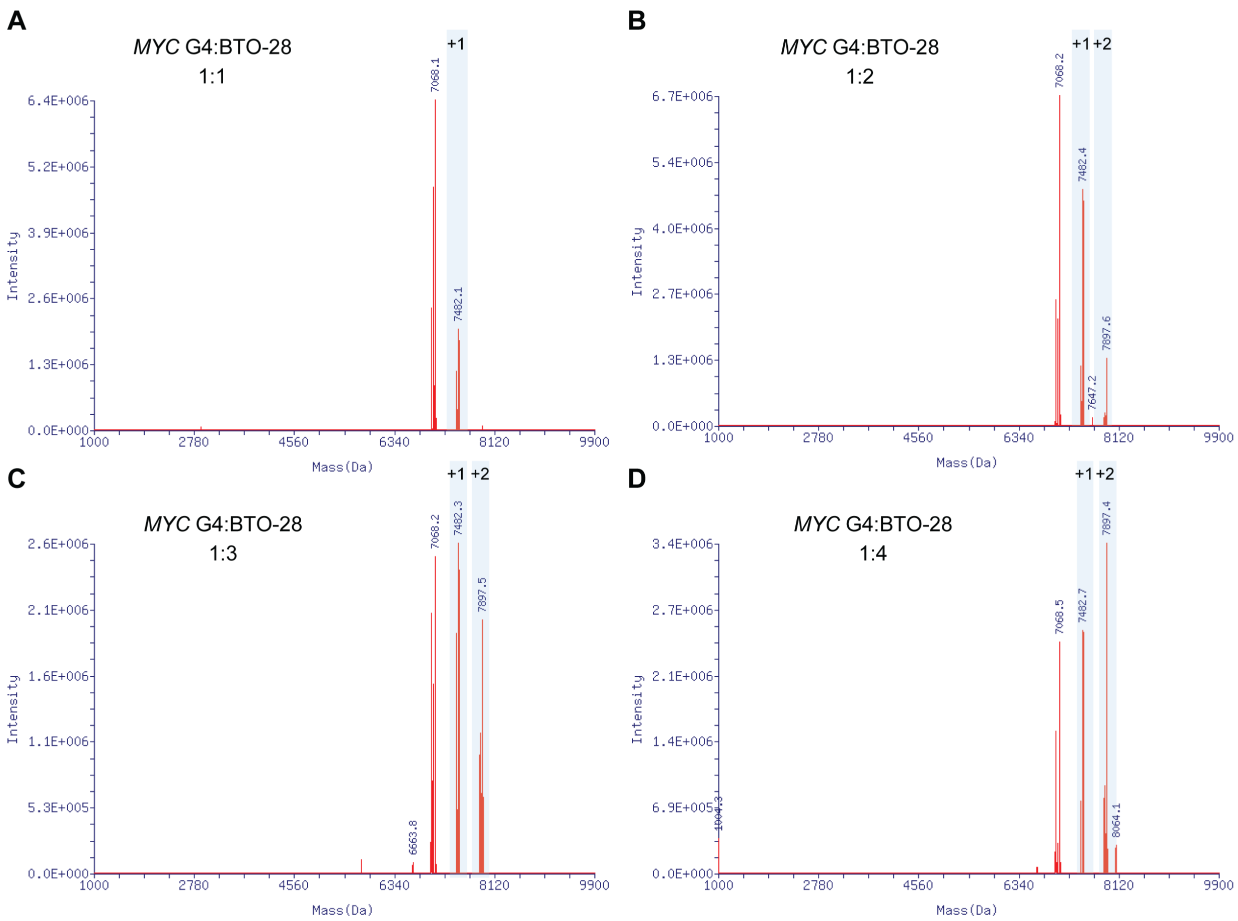

Figure S3. Negative ESI mass spectra of *MYC* G4 after incubation with varying ratios of BTO-28. Highlighted peaks correspond to G4 complexes bound to one or two ligands.

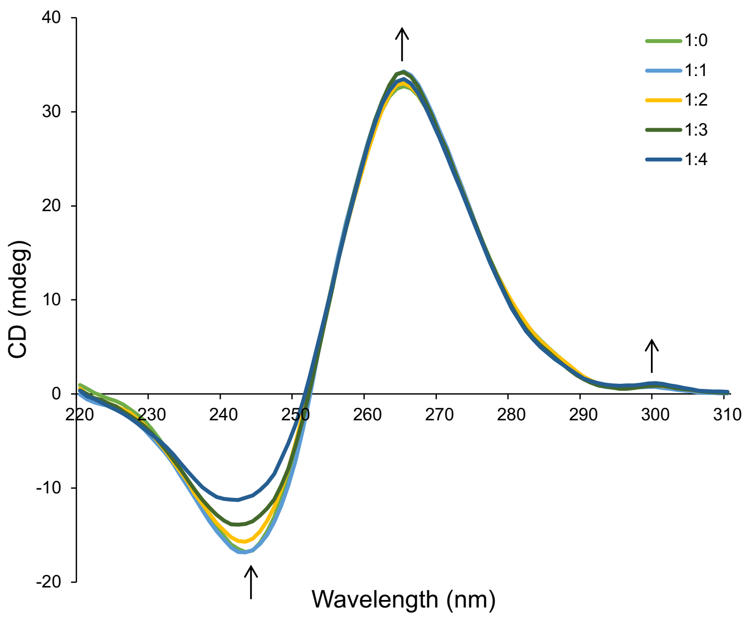

Figure S4. Impact of BTO-28 Binding on the CD Spectrum of *MYC* G4. Superimposed CD spectra of *MYC* G4 with varying DNA to BTO-28 ratios, recorded at 25 °C in 20 mM Tris-HCl, 80 mM KCl buffer, pH 7.4.

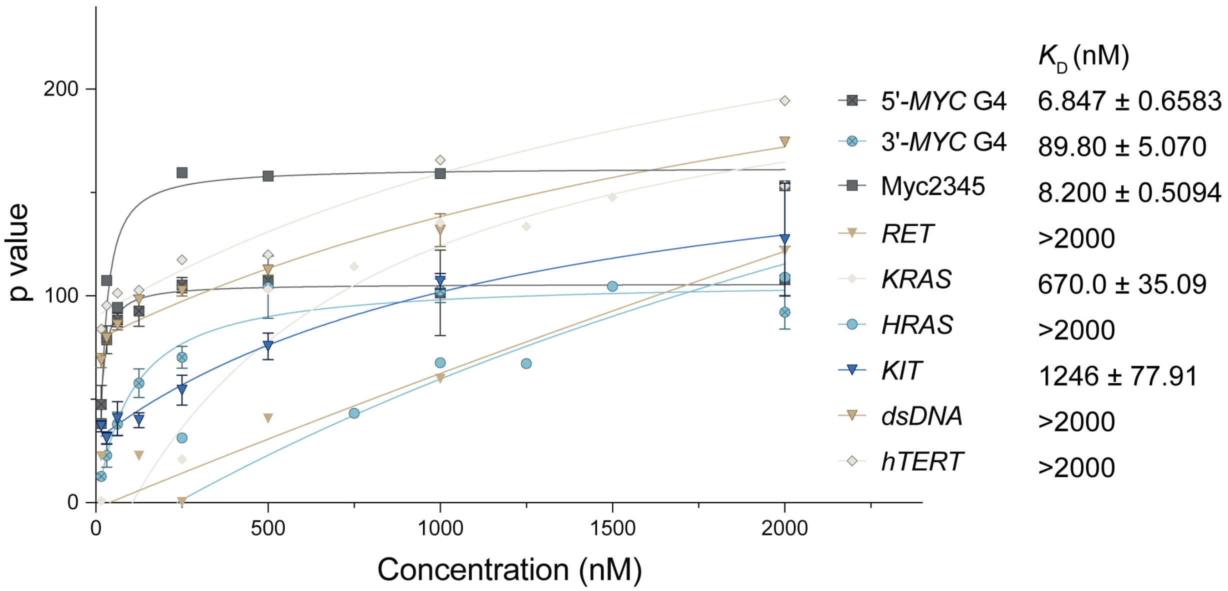

Figure S5. Binding curves determined by fluorescence polarization assays show that BTO-28 exhibits high affinity for *MYC* G-quadruplex structures.

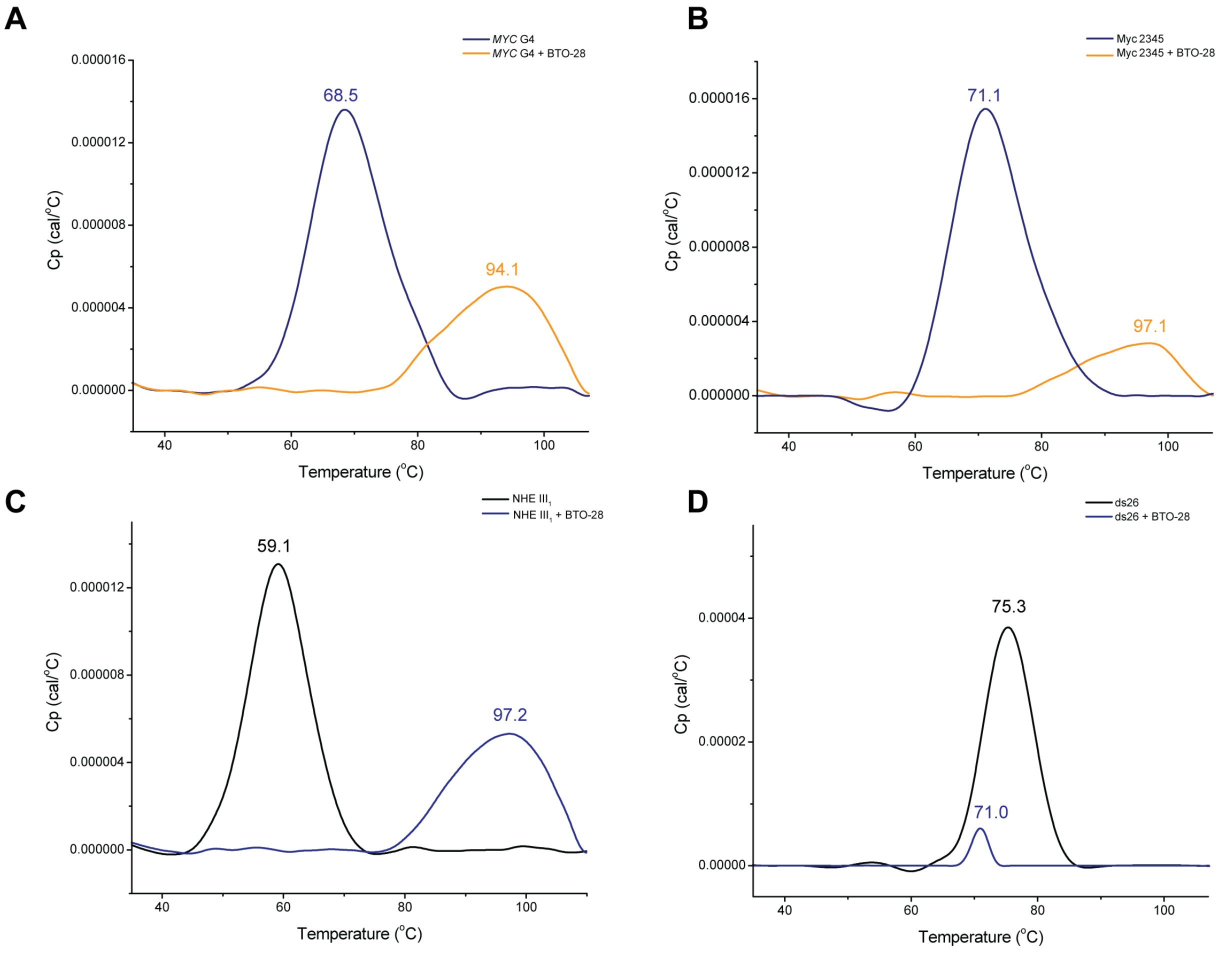

Figure S6. DSC melting temperatures of 50 μM (A) *MYC* G4, (B) Myc2345, (C) NHE III_1 and (D) dsDNA with or without BTO-28 (200 μM). Measured in 2 mM potassium phosphate, 8 mM KCl, pH 7.4.

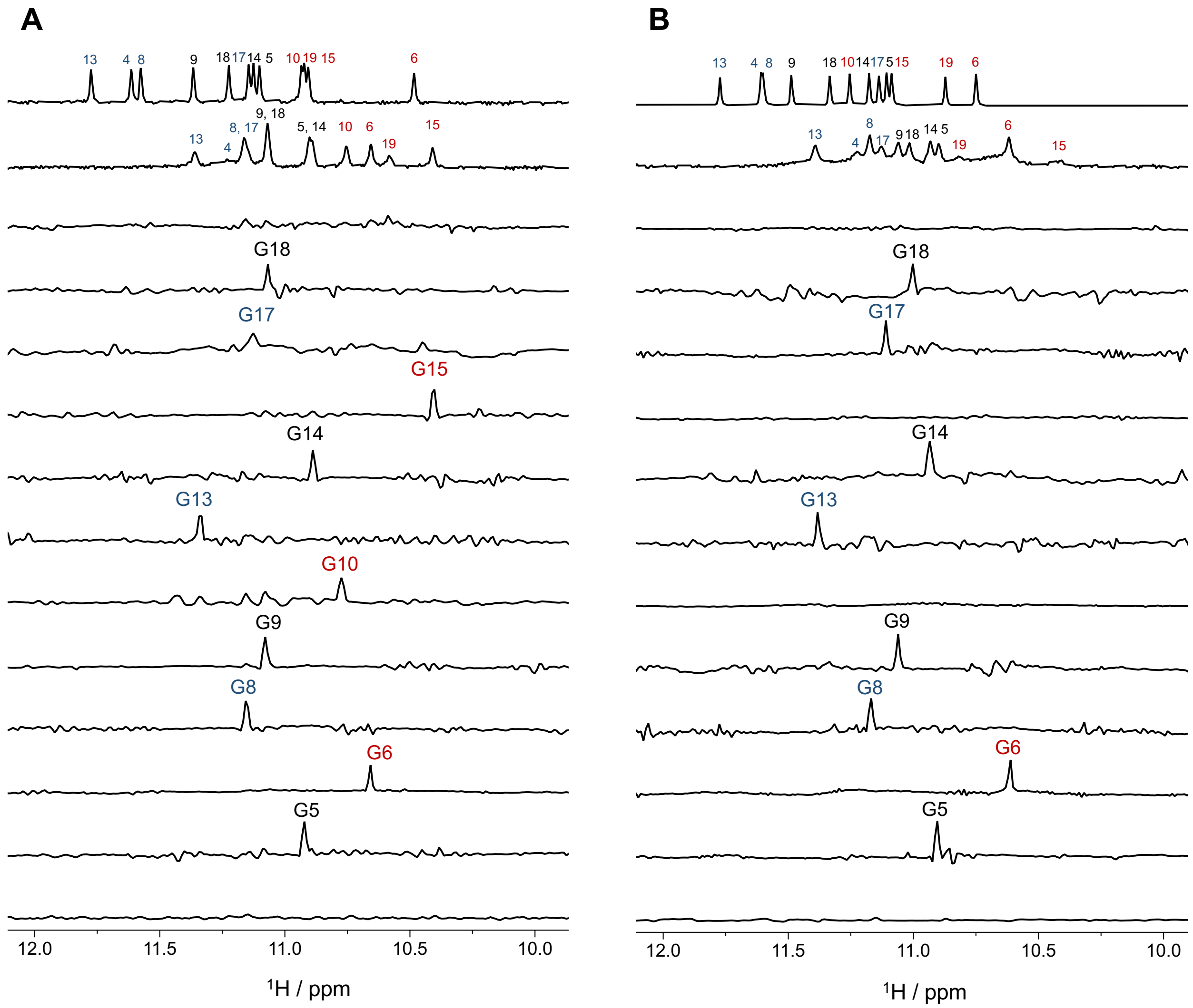

Figure S7. Imino proton assignments of (A) Benzothiazole–*MYC* G4 Complex and (B) Myc2345. Top: assignment of imino protons in the 1D ^{1}H NMR spectrum of *MYC* G4 in K^{+} solution. Bottom: Imino proton assignments of the benzothiazole–*MYC* G4 complex, determined using ^{1}H-^{15}N HSQC experiments on site-specifically labeled DNA. The imino protons from the 5′ G-tetrad are colored in blue, the middle G-tetrad in black, and the 3′ G-tetrad in red.

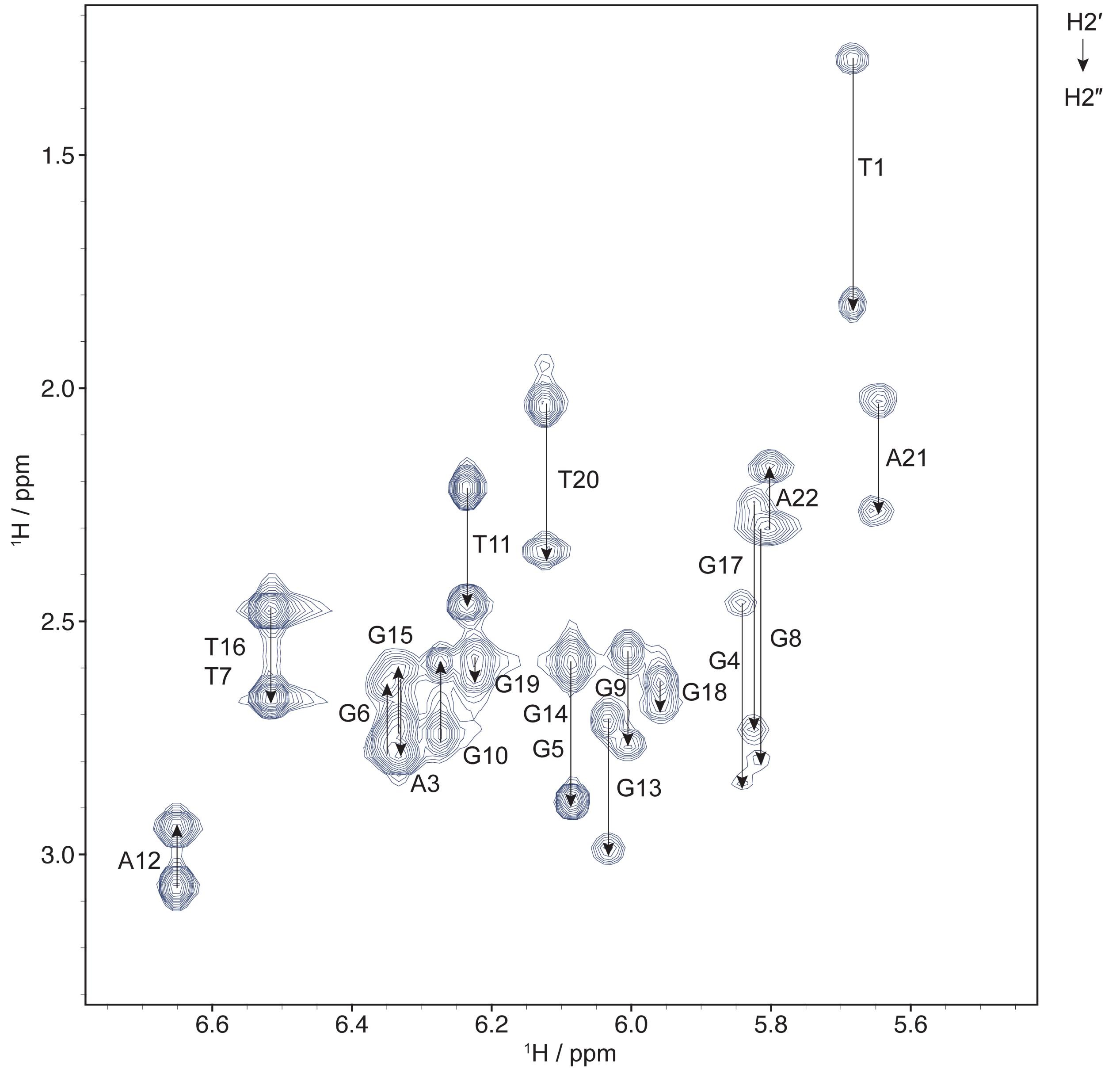

Figure S8. NMR analysis of benzothiazole–*MYC* G4 complex. TOCSY spectrum shows the H1′–H2′ and H1′–H2″ cross-peaks, with arrows indicating the H2′/H2″ assignments. Spectra were acquired in 10 mM K^+ buffer (pH 7.4) at 25°C and a DNA concentration of 700 μM.

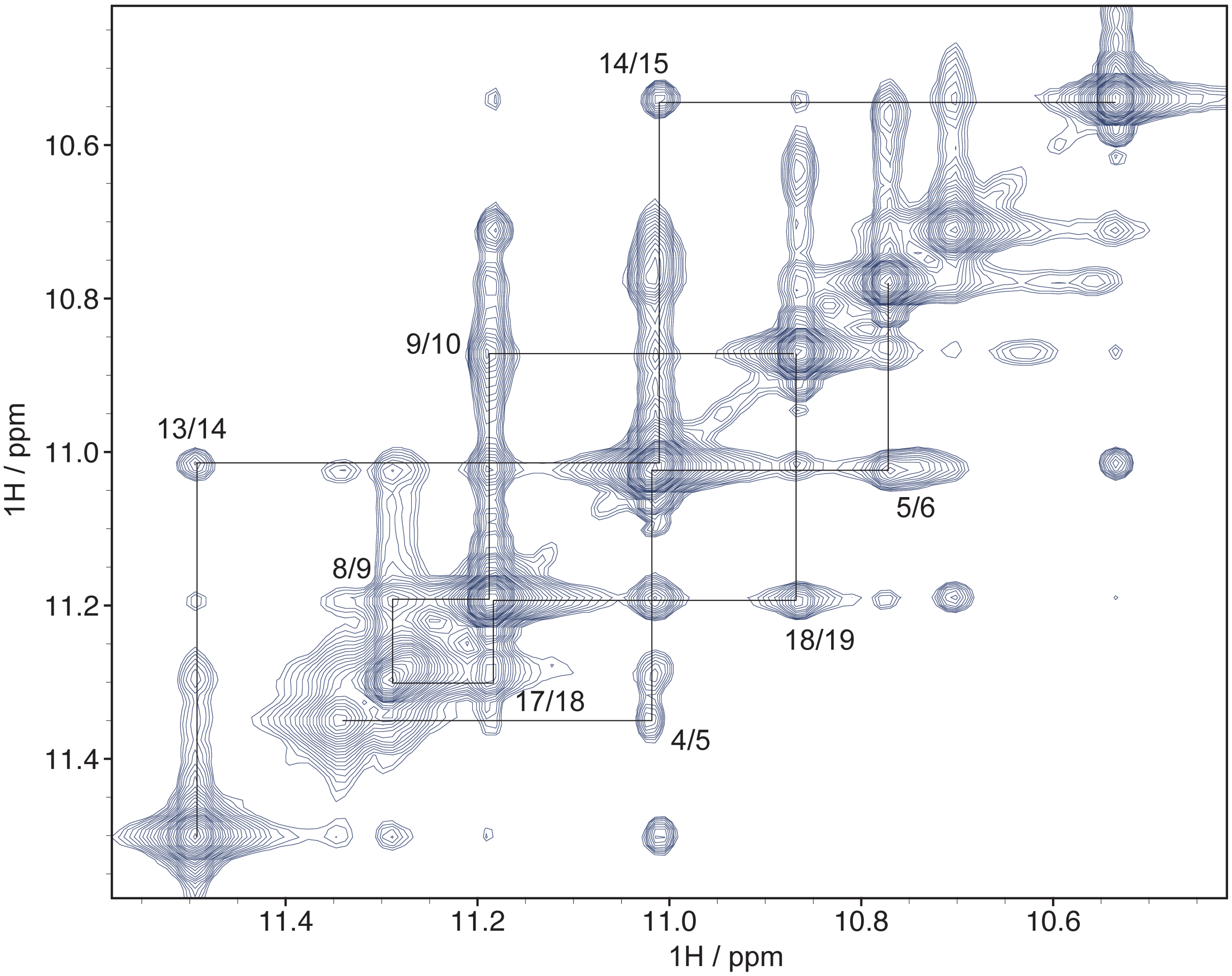

Figure S9. The expanded H1–H1 region of the NOESY spectrum reveals sequential imino–imino cross-peaks in individual G-strands. Spectra were acquired in 10 mM K^+ buffer (pH 7.4) at 25°C and a DNA concentration of 700 μM.

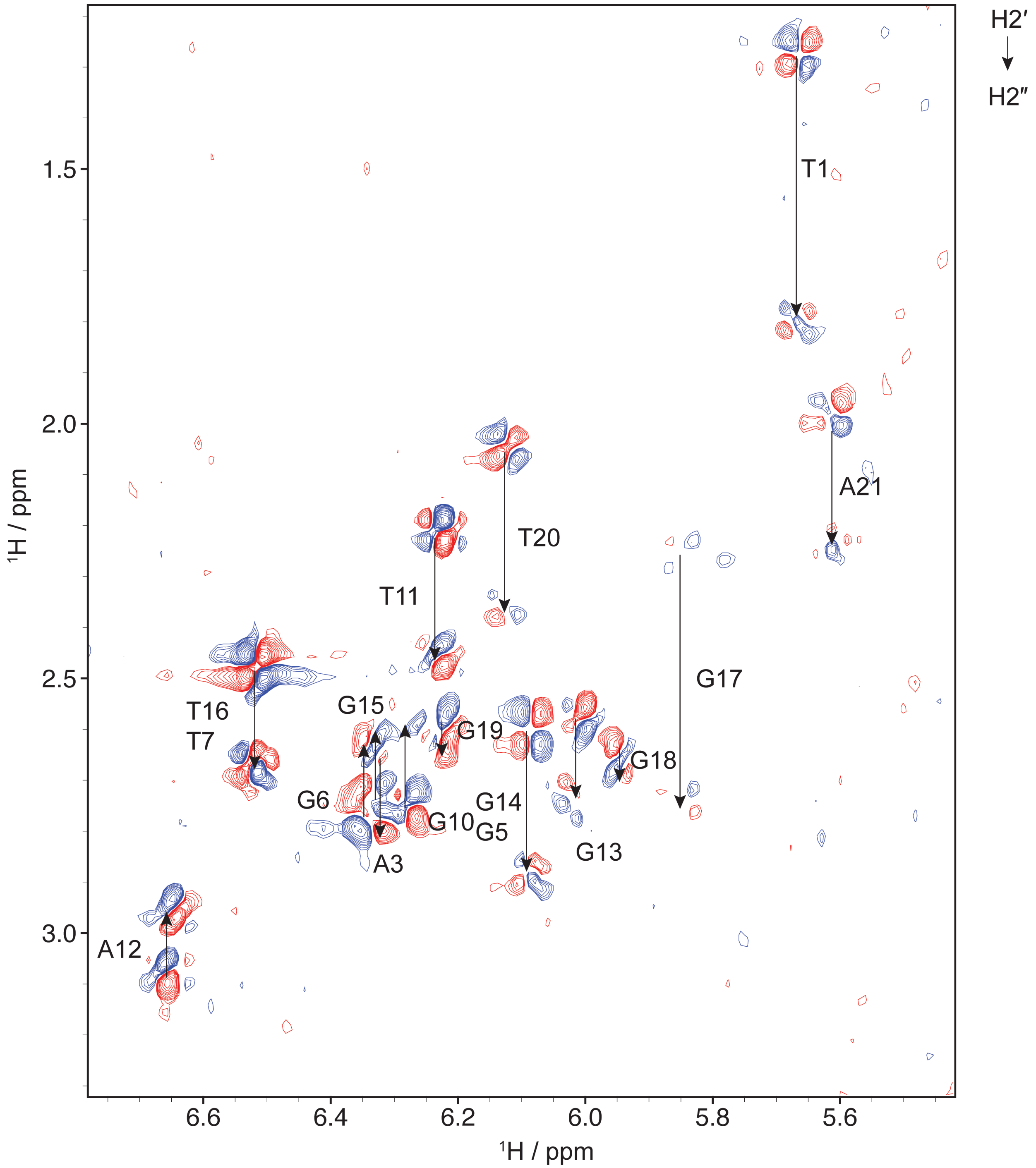

Figure S10. DQF-COSY spectrum of the benzothiazole–*MYC* G4 complex. Spectra were acquired in 10 mM K^+ buffer (pH 7.4) at 25°C and a DNA concentration of 700 µM.

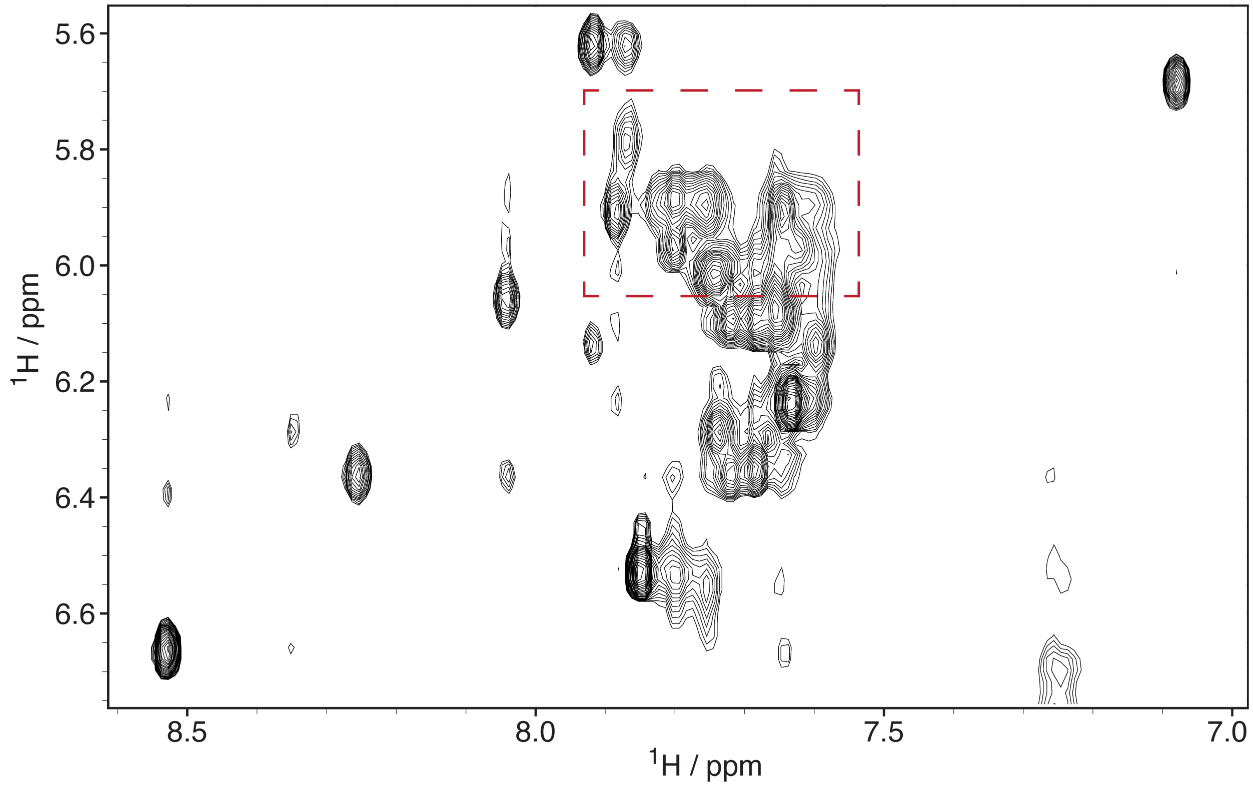

Figure S11. Expanded H8/H6–H1′ region of the 2D NOESY spectrum for the 2:1 Benzothiazole–*MYC* G4 complex, recorded in 100 mM K^+ solution at pH 7.4 and 25 °C. NOE peaks corresponding to multiple conformations are shown in the red dashed box.

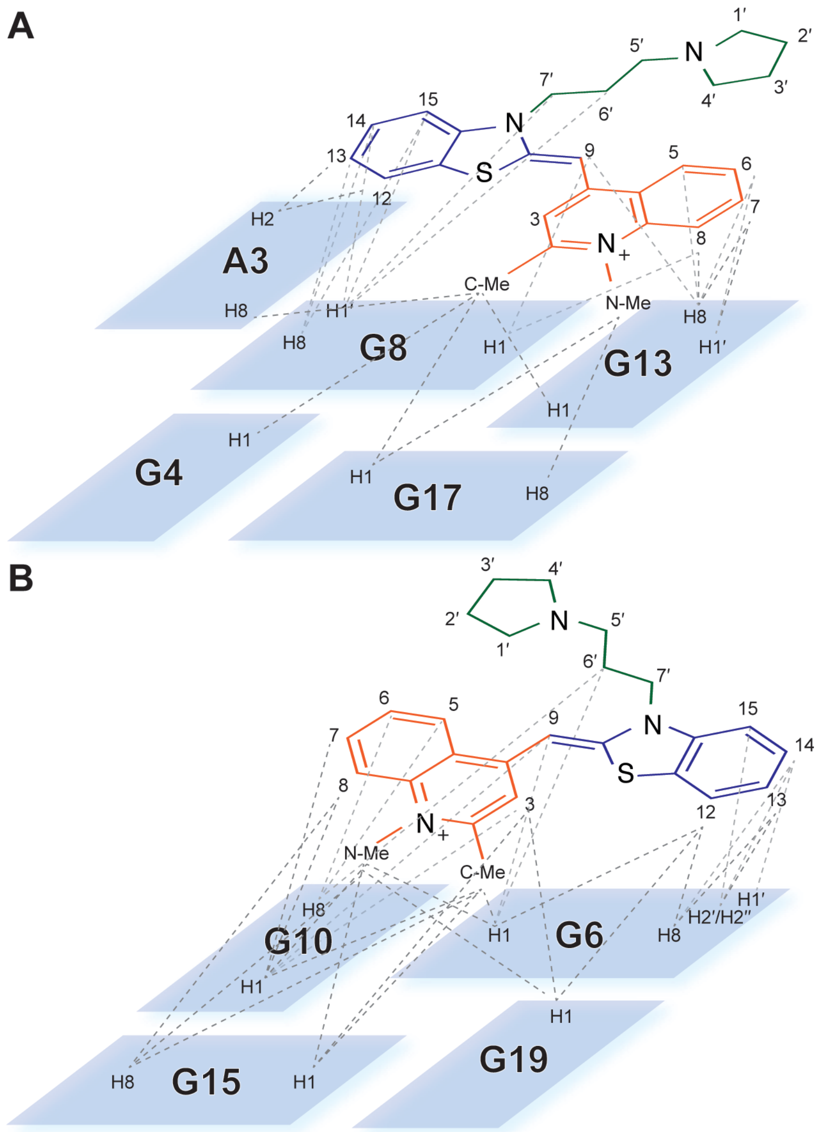

Figure S12. Representative inter-molecular interactions within the BTO-28–*MYC* G4 Complex at (A) 5′-end and (B) 3′-end.

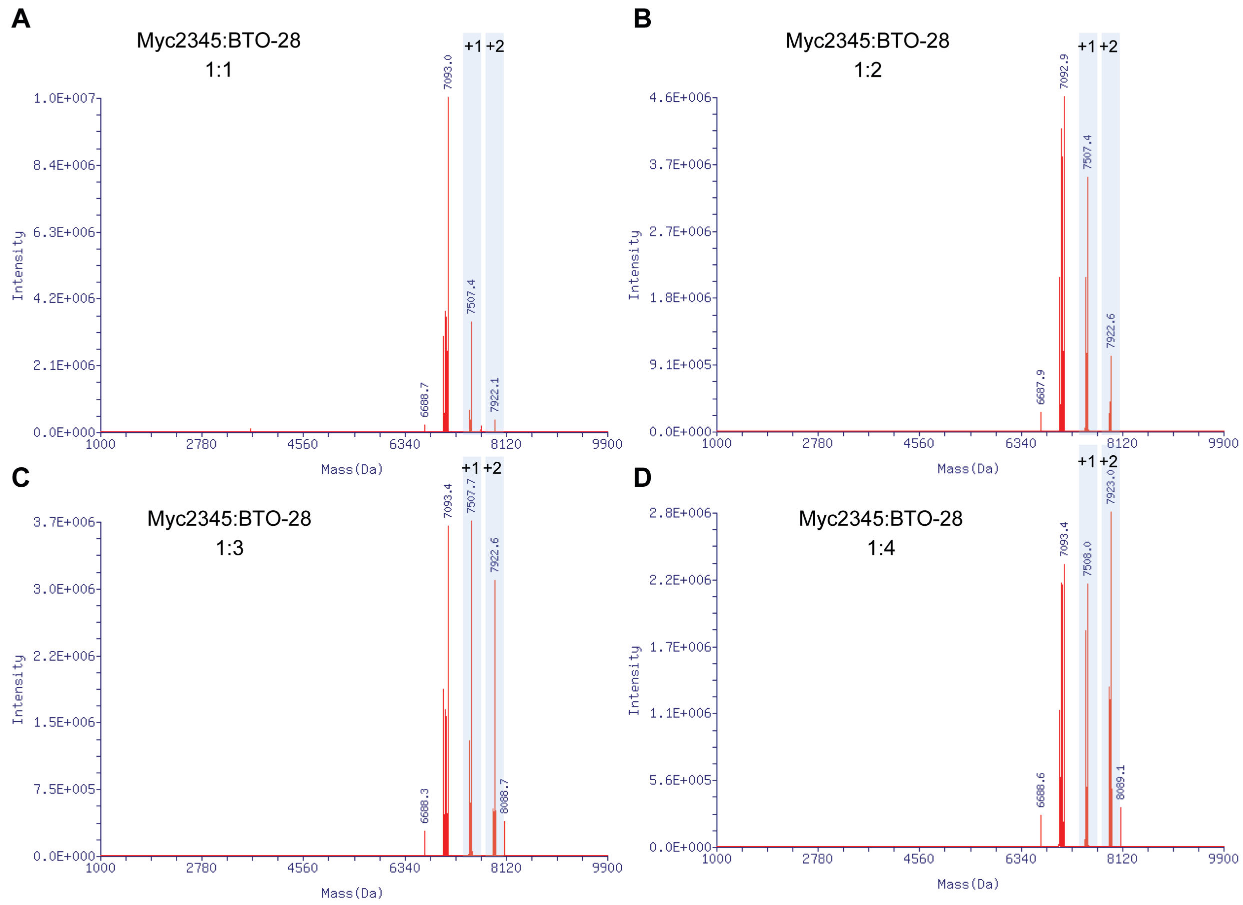

Figure S13. Negative ESI mass spectra of Myc2345 after incubation with varying ratios of BTO-28. Highlighted peaks correspond to G4 complexes bound to one or two ligands.

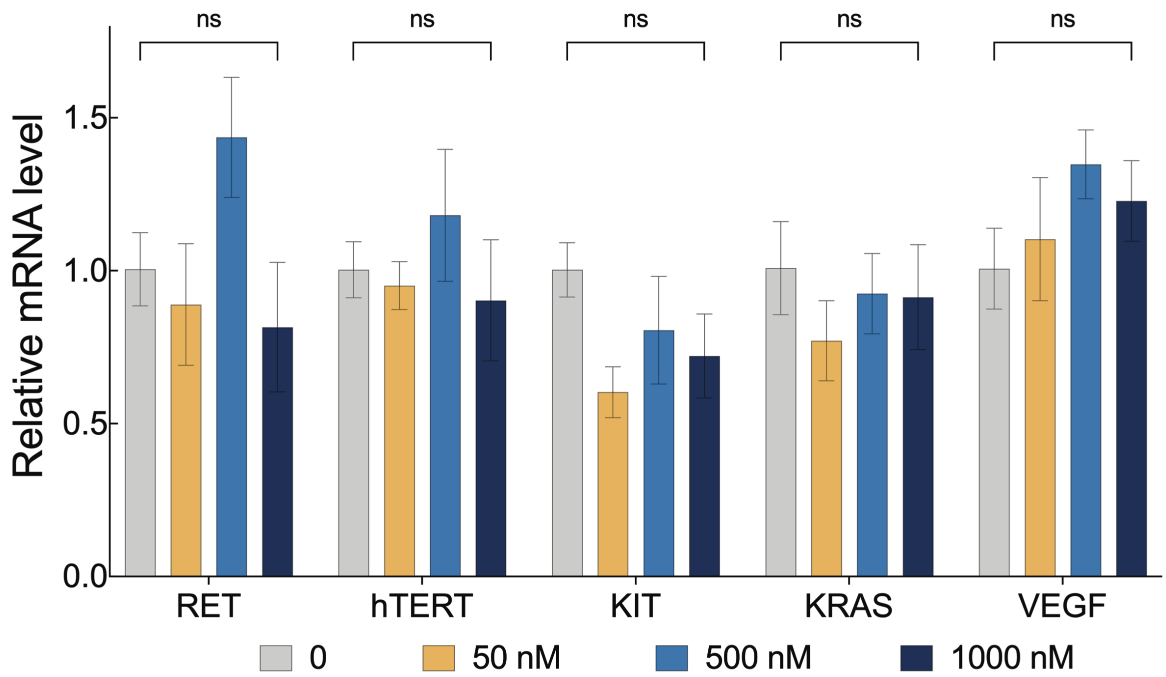

Figure S14. qTR-PCR analysis of G4-associated genes after treatment with BTO-28. ns, non-significant difference.

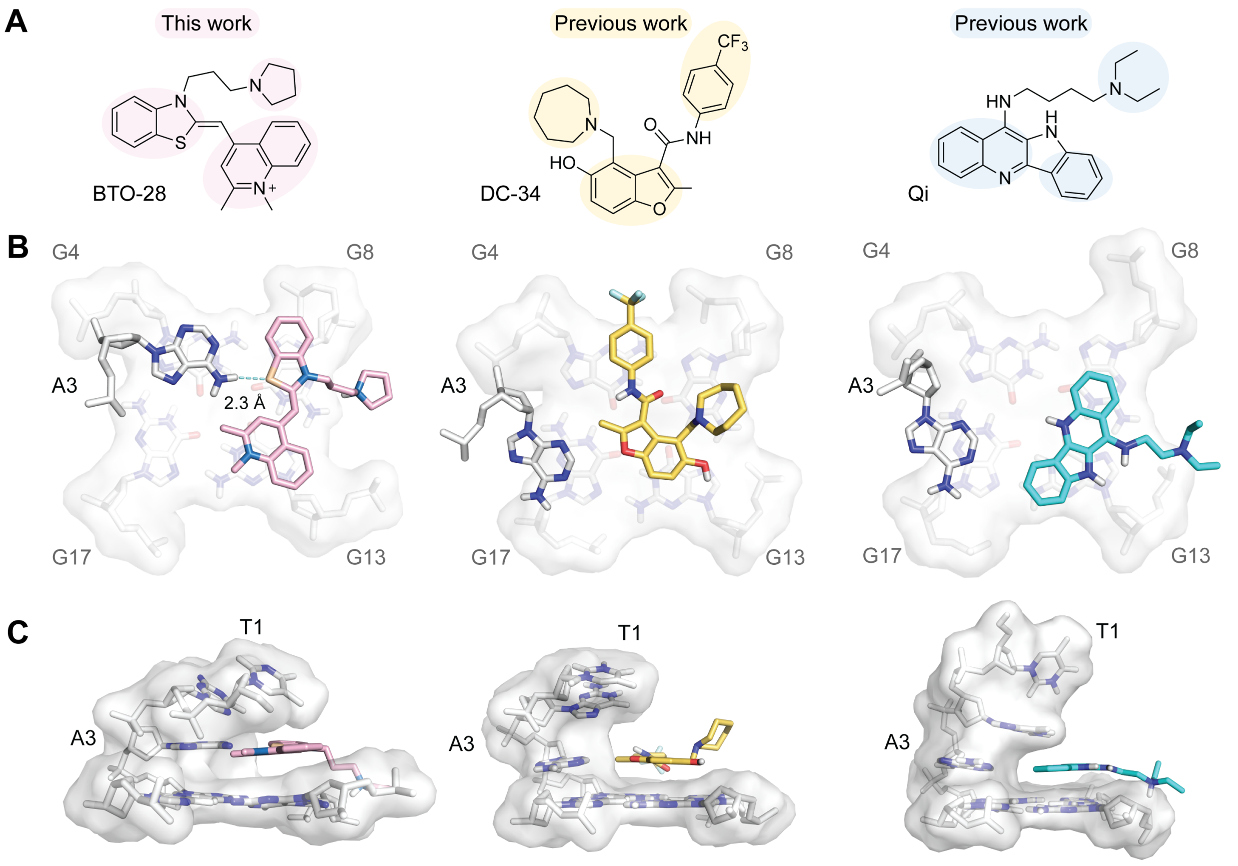

Figure S15. Comparison of structures of *MYC* G4 in complex with BTO-28 (PDB: 9L4E), DC-34 (PDB: 5W77) and Quindoline-i (PDB: 2L7V). (A) Chemical structures of the ligands. (B) 5′-sites of ligand–*MYC* G4 complexes (top view). (C) 5′-sites of ligand–*MYC* G4 complexes (side view).

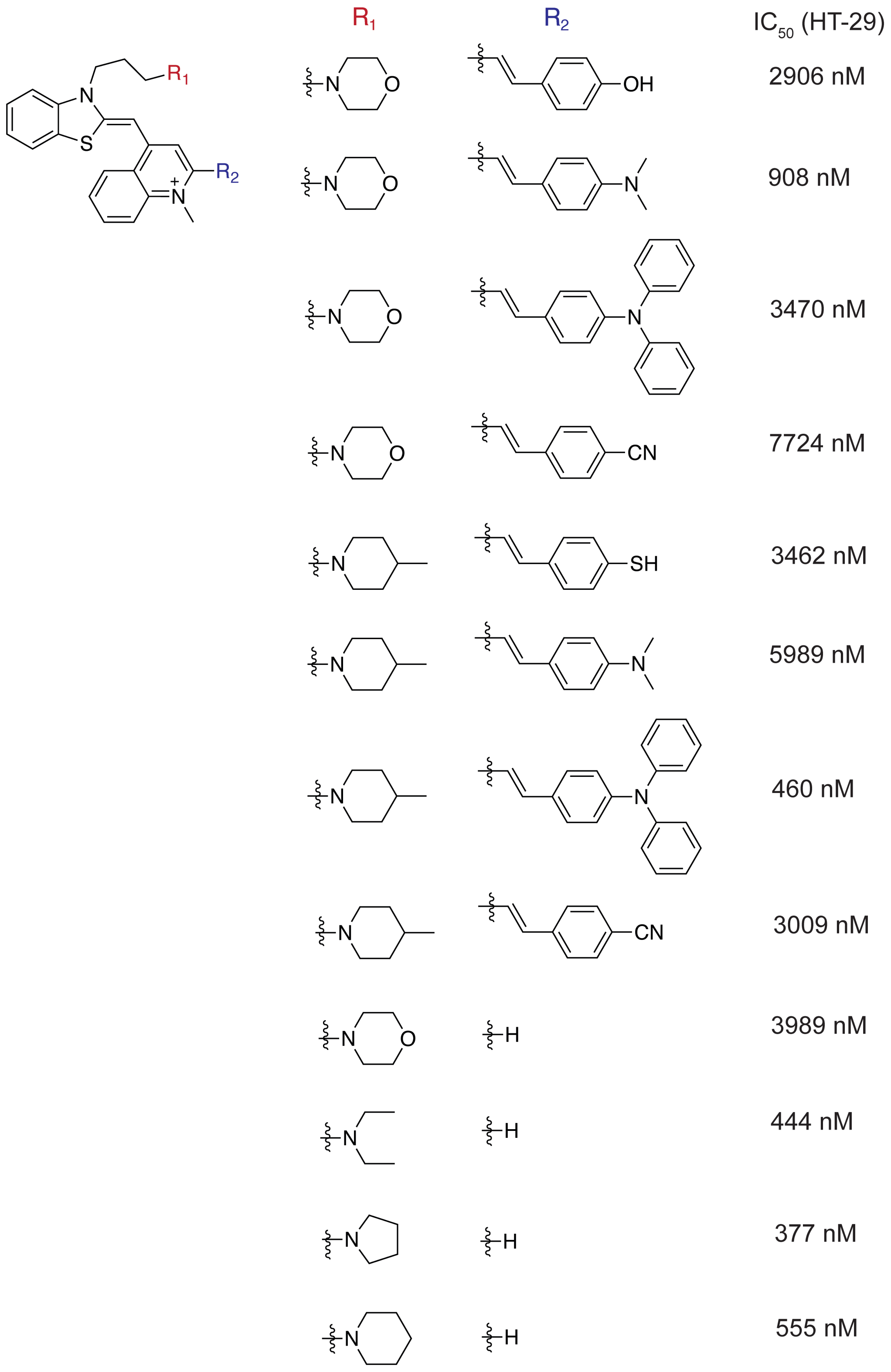

Figure S16. Analogs of BTO-28 were evaluated for cell viability assays in HT-29 cells.

2. Supplementary tables

Table S1. Oligonucleotides used in this study

Name	Sequence
**NMR Study and DSC**	
MYC G4	5′-TGAGGGTGGGTAGGGTGGGTAA-3′
Myc2345	5′-TGAGGGTGGGTAGGGTGGGGAA-3′
NHE III_1	5′-TGGGGAGGGTGGGGAGGGTGGGGAAGG-3′
dsDNA	5′-CAATCGGATCGAATTCGATCCGATTG-3′
**Fluorescence polarization**	
5′-*Cy5* *MYC* G4	5′-*Cy5* TGAGGGTGGGTAGGGTGGGTAA-3′
3′-*Cy5* *MYC* G4	5′-TGAGGGTGGGTAGGGTGGGTAA *Cy5*-3′
5′-*Cy5* dsDNA	5′-*Cy5* CAATCGGATCGAATTCGATCCGATTG-3′
5′-*Cy5* VEGF	5′-*Cy5* CGGGGCGGGCCGGGGGCGGGGT-3′
5′-*Cy5* KRAS	5′-*Cy5* AGGGCGGTGTGGGAAGAGGGAAGAGGGGGAGGCAG-3′
5′-*Cy5* RET	5′-*Cy5* GGGGCGGGGCGGGGCGGGGG-3′
5′-*Cy5* hTERT	5′-*Cy5* GGGTTAGGGTTAGGGTTAGGGTTAGGGTTAG-3′
5′-*Cy5* KIT	5′-*Cy5* GGGAGGGAGGGCGCTGGGAGGAGGG-3′
Taq **DNA polymerase stop assay**	
Taq-MYC	5′-TCCAACTATGTATACTA-TGAGGGTGGGTAGGGTGGGTAA-CACGCAATTGCTATAGTGAGTCGTATTA-3′
Taq-MYC-mutation[a]	5′-TCCAACTATGTATACTA-TGAAAGTAAGTAAAGTAAGTAA-CACGCAATTGCTATAGTGAGTCGTATTA-3′
Taq-FAM	5′-FAM-TAATACGACTCACTATAGCAATTGCGTG-3′
**qRT-PCR**	
MYC-P1-forward	CTTGGCGGGAAAAAGAACGG
MYC-P1-reverse	AGTTAGATAAAGCCCCGAAAACC
β-actin-forward	CTGGAAGCCTGAAGGTGACA
β-actin-reverse	AAGGGACTTCCTGTAACAACGCA
RET-forward	GCAGCATTGTTGGGGGACA
RET-reverse	CACCGGAAGAGGAGTAGCTG
hTERT-forward	AAATGCGGCCCCTGTTTCT
hTERT-reverse	CAGTGCGTCTTGAGGAGCA
cKIT-forward	CGTTCTGCTCCTACTGCTTCG
cKIT-reverse	CCCACGCGGACTATTAAGTCT
KRAS-forward	TGTGTCTCATATCAGGTTGACGA

Name	Sequence
**qRT-PCR**	
KRAS-reverse	CAAGAGTCGAGTGTGGTCTCA
VEGF-forward	GGAGGAGGGCAGAATCATCA
VEGF-reverse	CTTGGTGAGGTTTGATCCGC
**CHIP-qPCR**	
MYC-rev	CCGGCTTTTATACTCAGCGC
MYC-fwd	CCACCGGCCCTTTATAATGC
**Dual-luciferase assays**	
MYC-WT	5′-TGGGGAGGGTGGGGAGGGTGGGGAAGG-3′
MYC-Mut1[a]	5′-TGGGGAGGGTGAGGAGGGTGGGGAAGG-3′
MYC-Mut2[a]	5′-TAAAGAAAGTAAAGAAAGTAAAGAAGG-3′

[a]Mutations in the MYC sequence are highlighted in green.

Table S2. ^{1}H chemical shifts (ppm) of the 2:1 BTO-28-*MYC* G4 complex in 10 mM K^{+}.

	imino	H8/H6	H2/Me	H1′	H2′/H2″	H3′	H4′	H5′/H5″
T1	---	7.09	1.57	5.68	1.29/1.82	4.33	3.73	3.38/3.38
G2	---	7.23	---	5.20	2.09/1.94	4.64	3.96	3.59/3.71
A3	---	8.27	8.04	6.33	2.79/2.79	4.98	4.29	4.00/4.00
G4	11.30	7.89	---	5.84	2.46/2.85	4.96	4.44	4.20/4.28
G5	11.00	7.65	---	6.09	2.58/2.88	4.99	4.51	4.20/4.22
G6	10.80	7.72	---	6.35	2.76/2.64	5.16	4.58	4.31/4.33
T7	---	7.86		6.52	2.58/2.58	4.98	4.41	
G8	11.30	7.80	---	5.82	2.29/2.80	5.05	4.40	4.31/4.40
G9	11.20	7.75	---	6.01	2.57/2.76	5.03	4.41	4.24/4.17
G10	10.90	7.73	---	6.27	2.74/2.59	5.04	4.45	4.25/4.25
T11	---	7.65	1.95	6.24	2.21/2.46	4.73	3.92	3.79/3.77
A12	---	8.54	8.35	6.65	3.07/2.94	5.18	4.58	4.19/4.29
G13	11.50	8.05	---	6.03	2.71/2.99	4.99	4.52	4.19/4.22
G14	11.00	7.66	---	6.09	2.60/2.89	5.02	4.52	4.30/4.30
G15	10.50	7.68	---	6.33	2.72/2.60	5.15	4.58	4.31/4.34
T16	---	7.86	2.00	6.52	2.48/2.66	5.09	4.58	4.26/4.34
G17	11.30	7.74	---	5.83	2.25/2.73	5.06	4.42	4.23/4.31
G18	11.20	7.80	---	5.96	2.63/2.68	5.08	4.51	4.26/4.18
G19	10.70	7.63	---	6.22	2.59/2.62	5.08	4.54	4.25/4.30
T20	---	7.60	1.93	6.13	2.04/2.35	4.80	4.28	4.17/4.10
A21	---	7.94	7.27	5.65	2.03/2.26	4.64	3.84	3.76/3.76
A22	---	7.90	7.31	5.80	2.30/2.17	4.41	3.76	3.66/3.66

Table S3. Intramolecular NOE interactions of *MYC* G4 in complex with BTO-28 involving the 5′-terminal residues T1-G4. Cross-peak intensities are categorized as strong (S), medium (M), weak (W) or very weak (V).

**T1**		**H1′**	**H2′**	**H2″**	**H3′**	**H5′**
**G2**	**H8**	V	M	W	M	W
	**H4′**			M		
	**H5′**			S		
	**H5″**			M		

**G2**		**H8**	**H1′**	**H2′**	**H2″**	**H3′**	**H4′**	**H5′**
**T1**	**H6**						W	
	**H1′**	V					V	W
	**H3′**				W			
**A3**	**H2**	M						
	**H8**	W	V	S	M	S	V	

**A3**		**H8**	**H2**	**H1′**	**H2″**
**G4**	**H1′**		M		
	**H5′**	V		M	M
	**H5″**				M

**G4**		**H1**	**H8**	**H1′**	**H2′**	**H2″**	**H3′**	**H4′**	**H5′**
**A3**	**H8**								V
	**H2**			M					
	**H1′**								M
**G5**	**H1**	M							
	**H8**		M	M	S	S	W	W	
	**H1′**					W			
	**H4′**			W					
	**H5′**			M		S			
	**H5″**			W		M			
**G17**	**H1**	M	M						
**G18**	**H1**		W		V	V			

Table S4. Intramolecular NOE interactions of *MYC* G4 in complex with BTO-28 involving the 3′-terminal residues G19-A23. Cross-peak intensities are categorized as strong (S), medium (M), weak (W) or very weak (V).

**G19**		**H1**	**H8**	**H1′**	**H2′**	**H2″**	**H3′**	**H4′**	**H5′**	**H5″**
**G5**	**H1**	M								
	**H8**	M								
	**H2″**	W								
**G6**	**H1**	M								
**G15**	**H1**	W	W							
**G18**	**H1**	M								
	**H8**		M							
	**H1′**		M					M	S	M
**T20**	**H6**			M	S	S	W	W		
	**H5′**					S				
	**H5″**					W				
	**Me**				S	W				

**T20**		**H6**	**H1′**	**H2′**	**H2″**	**H3′**	**H4′**	**H5′**	**H5″**	**Me**
**G19**	**H8**	M						V		
	**H1′**						V	M	V	V
	**H4′**									V
**A21**	**H2**		V							
	**H8**		V		W	V	V			
	**H1′**		V							
	**H4′**				V					
	**H5′**				V					
**A22**	**H8**		V							

**A21**		**H2**	**H1′**	**H2′**	**H2″**	**H3′**	**H4′**	**H5′**
**T20**	**H1′**					V	V	V
	**H4′**							M
**A22**	**H2**	V	V					
	**H8**		V	W	M	V	V	
	**H5′**				S			

**A22**		**H4′**	**H5′**
**A21**	**H1′**	W	V
	**H2″**		S

Table S5. ^{1}H chemical shifts (ppm) of BTO-28 in the 2:1 BTO-28-*MYC* G4 complex in 10 mM K^{+}.

N-Me	C-Me	H3	H5	H6	H7	H8	H9	H12
3.93	3.65	7.80	7.80	7.61	7.94	7.98	6.09	7.58
H13	H14	H15	H1′	H2′	H4′	H6′	H7′	
7.48	7.74	7.66	2.36	1.95	2.35	1.95	4.58	

Table S6. Observed intermolecular NOE cross-peaks between *MYC* G4 5′-end and BTO-28. The intensity of the NOE peaks is showed by ‘S’ (strong, 1.8-2.9 Å), ‘M’ (medium, 1.8-3.5 Å), ‘W’ (weak, 1.8-5.0 Å) or ‘V’ (very weak, 1.8-6.0 Å) respectively.

		BTO-28													
		**H15**	**H14**	**H13**	**H12**	**H9**	**H5**	**H6**	**H7**	**H8**	**H3**	**N-Me**	**C-Me**	**7′**	**6′**
**T1**	**H6**						W		W		W		W		
	**Me**			W									W		
**G2**	**H8**										W		W		
**A3**	**H2**			W	M										
	**H8**												W		
**G4**	**H1**												W		
**G8**	**H1**					M				V					
	**H8**	M	W	M											
	**H1′**	M	W											M	M
**G13**	**H1**												M		
	**H8**					W	M	S	M	W					
	**H1′**							W	S						
**G17**	**H1**											W	W		
	**H8**											V			

Table S7. Observed intermolecular NOE cross-peaks between *MYC* G4 3′-end and BTO-28. The intensity of the NOE peaks is showed by ‘S’ (strong, 1.8-2.9 Å), ‘M’ (medium, 1.8-3.5 Å), ‘W’ (weak, 1.8-5.0 Å) or ‘V’ (very weak, 1.8-6.0 Å) respectively.

		BTO-28														
		**H15**	**H14**	**H13**	**H12**	**H9**	**H5**	**H6**	**H7**	**H8**	**H3**	**N-Me**	**C-Me**	**7′**	**6′**	**1′**
**G6**	**H1**				W	M						V	W	W		
	**H8**		M	M	M											
	**H1′**	M	M													M
	**H2′**		M	M												
	**H2″**	M	S													M
**G10**	**H1**					W			M	M	W	W	W			
	**H8**						M	W							M	M
	**H2′**						M	M	W							
	**H2″**						W	M	M							
**G15**	**H1**										W	W	M			
	**H8**									W		M	V			
**G19**	**H1**				W						W	V				
**T20**	**H1′**			V	W											
**A21**	**H2**			V	V											

3. Synthesis and characterization

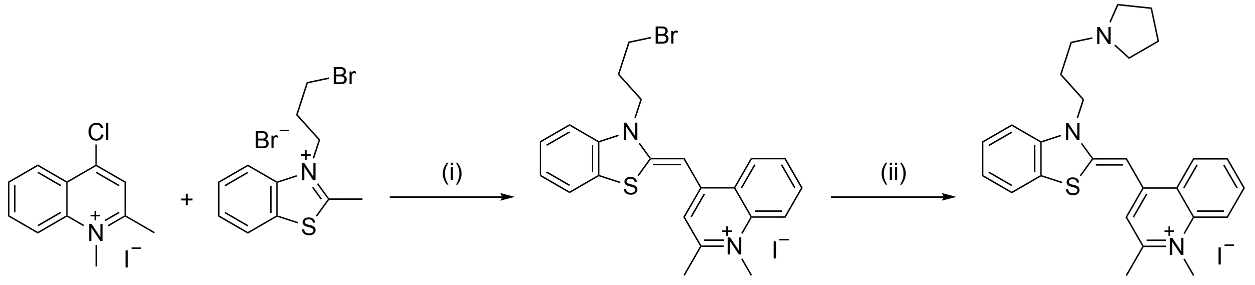

1 2 3 **BTO-28**

Scheme S1. Synthetic path of **BTO-28**. Reagents and conditions: (i) methanol, 40 °C, 24 h; (ii) pyrrolidine, potassium iodide, 40°C, 24 h.

BTO-28 was prepared according to literature procedures.[1] Intermediate **3** was obtained by the reaction using 4-chloro-1,2-dimethylquinolin-1-ium iodide (**1**, 2.0 mmol, 1.0 eq.) and 3-(3-bromopropyl)-2-methylbenzo[d]thiazole-3-ium bromide (**2**, 2.0 mmol, 1.0 eq.) for 24 h at 40 °C in methanol solution (yield = 80%). BTO-28 was further obtained by reacting intermediate **3** (2.0 mmol, 1.0 eq.) with 10.0 mmol (5.0 eq.) of pyrrolidine and statured potassium iodide for 24 h at 40 °C (yield = 61%).

^{1}H NMR (400 MHz, [D$_6$]DMSO, ppm): δ 8.80 (d, J = 8.3 Hz, 1H), 8.24 (d, J = 8.7 Hz, 1H), 8.04 (t, J = 7.1 Hz, 2H), 7.80 (t, J = 7.9 Hz, 2H), 7.63 (t, J = 8.2 Hz, 1H), 7.48–7.39 (m, 2H), 6.87 (s, 1H), 4.67 (t, J = 7.3 Hz, 2H), 4.12 (s, 3H), 3.57 (s, 2H), 3.43 (s, 2H), 3.05 (s, 2H), 2.92 (s, 3H), 2.19 (dt, J = 13.3, 6.7 Hz, 2H), 2.02 (s, 2H), 1.87 (s, 2H).

^{13}C NMR (100MHz, [D$_6$]DMSO, ppm): 159.28, 154.54, 148.33, 140.62, 139.51, 133.55, 128.47, 126.72, 125.50, 124.69, 124.29, 123.90, 123.21, 118.83, 113.24, 110.97, 100.00, 87.08, 53.89, 52.45, 44.18, 40.64, 40.35, 40.22, 40.01, 39.80, 39.59, 39.38, 37.63, 26.45, 23.63, 23.35.

HRMS (ESI): m/z calcd for $C_{26}H_{30}N_3S^+$: 416.2155; found: 416.2177.

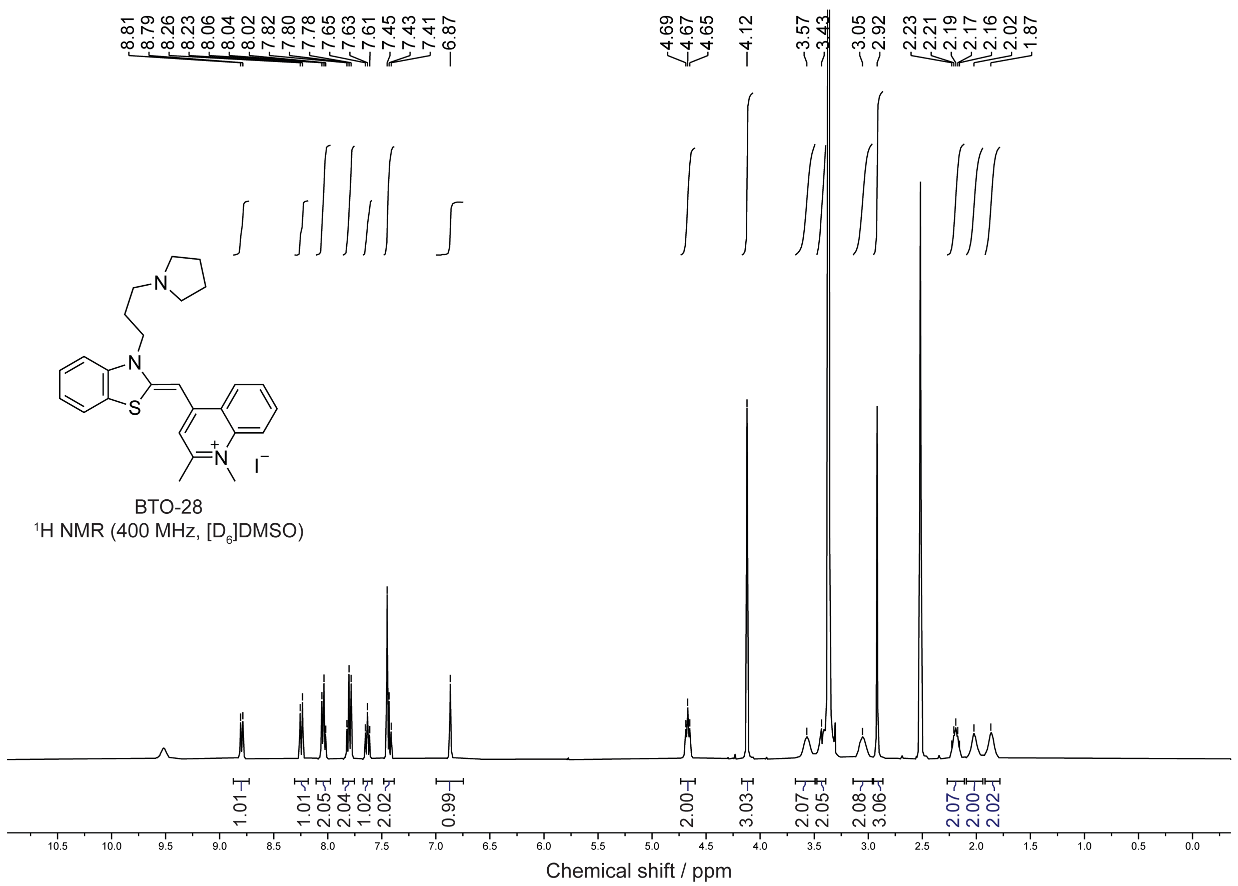

Figure S17. ^{1}H NMR spectrum of **BTO-28**.

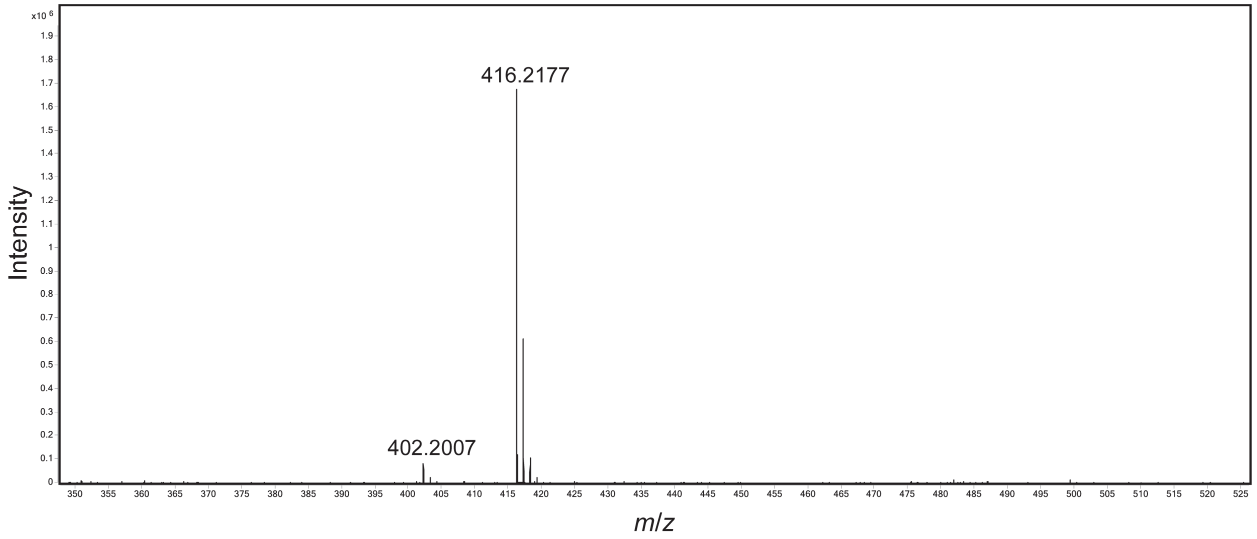

Figure S18. High resolution mass spectrometry of **BTO-28**.

Reference

[1] W. Long, Y.-J. Lu, K. Zhang, X.-H. Huang, J.-Q. Hou, S.-Y. Cai, Y. Li, X. Du, L. G. Luyt, W.-L. Wong, C.-F. Chow, "Boosting the turn-on fluorescent signaling ability of thiazole orange dyes: The effectiveness of structural modification site and its unusual interaction behavior with nucleic acids" *Dyes Pigm.* **2018**, *159*, 449-456.
